# Bone turnover change after randomized switch from tenofovir disoproxil to tenofovir alafenamide fumarate in men with HIV

**DOI:** 10.1097/QAD.0000000000003811

**Published:** 2024-02-01

**Authors:** Amelia E.B. Moore, James E. Burns, Deirdre Sally, Ana Milinkovic, Georgios Krokos, Joemon John, Christopher Rookyard, Alessandro Borca, Erica R.M. Pool, Anna Tostevin, Alyss Harman, Dwight S. Dulnoan, Richard Gilson, Alejandro Arenas-Pinto, Gary J.R. Cook, John Saunders, David Dunn, Glen M. Blake, Sarah L. Pett

**Affiliations:** a Department of Cancer Imaging, School of Biomedical Engineering and Imaging Sciences, King's College London, St. Thomas’ Hospital; b Osteoporosis Unit, Guy's and St Thomas’ NHS Foundation Trust; c Centre for Clinical Research in HIV and Sexual Health, Institute for Global Health, University College London; d Mortimer Market Centre, Central and North West London NHS Foundation Trust, London; e Department of Biomedical Engineering, School of Biomedical Engineering and Imaging Sciences, King's College London, St. Thomas’ Hospital, London, UK.

**Keywords:** bone density, bone turnover, HIV, PET/computed tomography, tenofovir

## Abstract

**Objective:**

Bone loss in people with HIV (PWH) is poorly understood. Switching tenofovir disoproxil fumarate (TDF) to tenofovir alafenamide (TAF) has yielded bone mineral density (BMD) increases. PETRAM (NCT#:03405012) investigated whether BMD and bone turnover changes correlate.

**Design:**

Open-label, randomized controlled trial.

**Setting:**

Single-site, outpatient, secondary care.

**Participants:**

Nonosteoporotic, virologically suppressed, cis-male PWH taking TDF/emtricitabine (FTC)/rilpivirine (RPV) for more than 24 weeks.

**Intervention:**

Continuing TDF/FTC/RPV versus switching to TAF/FTC/RPV (1 : 1 randomization).

**Main outcome measures:**

:[^18^F]NaF-PET/CT for bone turnover (standardized uptake values, SUV_mean_) and dual-energy x-ray absorptiometry for lumbar spine and total hip BMD.

**Results:**

Thirty-two men, median age 51 years, 76% white, median duration TDF/FTC/RPV 49 months, were randomized between 31 August 2018 and 09 March 2020. Sixteen TAF:11 TDF were analyzed. Baseline-final scan range was 23–103 (median 55) weeks. LS-SUV_mean_ decreased for both groups (TAF -7.9% [95% confidence interval -14.4, -1.5], TDF -5.3% [-12.1,1.5], *P* = 0.57). TH-SUV_mean_ showed minimal changes (TAF +0.3% [-12.2,12.8], TDF +2.9% [-11.1,16.9], *P* = 0.77). LS-BMD changes were slightly more favorable with TAF but failed to reach significance (TAF +1.7% [0.3,3.1], TDF -0.3 [-1.8,1.2], *P* = 0.06). Bone turnover markers decreased more with TAF ([CTX -35.3% [-45.7, -24.9], P1NP -17.6% [-26.2, -8.5]) than TDF (-11.6% [-28.8, +5.6] and -6.9% [-19.2, +5.4] respectively); statistical significance was only observed for CTX (*P* = 0.02, P1NP, *P* = 0.17).

**Conclusion:**

Contrary to our hypothesis, lumbar spine and total hip regional bone formation (SUV_mean_) and BMD did not differ postswitch to TAF. However, improved LS-BMD and CTX echo other TAF-switch studies. The lack of difference in SUV_mean_ may be due to inadequate power.

## Introduction

The widespread use of lifelong contemporary combination antiretroviral therapy (ART) regimens, many of which include the nucleotide reverse transcriptase inhibitor (NRTI), tenofovir disoproxil fumarate (TDF) [1], has transitioned HIV into a chronic disease with normal life expectancy [2]. However, bone loss and its sequelae (fractures) [2] are increasingly recognized as important comorbidities in the aging population of people with HIV (PWH). Moreover, it is likely that bone loss is underestimated in this population, as the tools for detection are insensitive, not widely available, or not utilized. Although the mechanism of bone loss is poorly understood, plasma bone turnover markers (BTMs) suggest uncoupling of bone resorption and formation via a treatment effect on bone cells [3].

Although there are likely several contributing factors (age-associated, vitamin D deficiency, corticosteroid exposure, HIV-associated perturbation of bone turnover), TDF-containing regimens have been associated with greater bone loss [4]. Switching from a TDF-containing regimen to one containing tenofovir alafenamide (TAF), a phosphonoamidate prodrug of tenofovir, has been associated with bone mineral density (BMD) increases as measured by dual energy X-ray absorptiometry (DXA), which suggests a reversal of subclinical bone loss [5–9].

Methods to identify fracture risk and BMD loss in PWH are confined to the Frax score [10], which has not been specifically validated in PWH, and DXA, which only provides two-dimensional impressions of fracture risk at the hip and spine. In the HIV field, bone turnover markers (BTMs) and bone biopsy are used as research tools. The former provides a global assessment of bone turnover but lacks the ability to differentiate changes between bone sites, and the latter is invasive and only provides information on the anatomical site biopsied, which may not be representative.

Novel radiological platforms such as PET/computed tomography with radiolabeled sodium fluoride ([^18^F]NaF-PET/CT) can offer noninvasive quantitative assessment of bone turnover at specified sites [11–14]. Bone turnover is the cyclical process of bone resorption and formation. [^18^F]NaF-PET/CT measures the bone metabolic activity relative to a uniform whole-body distribution of the radiolabeled tracer (termed standardized uptake value [SUV_mean_]), to determine if there is increased or decreased bone turnover at the analyzed sites. A decrease in SUV_mean_ is correlated with a reduction in bone turnover. This technique has been validated in non-HIV cohorts and in those with metabolic bone diseases [15–22].

The aim of the PETRAM study is to use [^18^F]NaF-PET/CT to explore bone turnover at the hip and lumbar spine in nonosteoporotic males, aged 40–65 years, living with HIV, randomized to either switch to a TAF-based regimen (TAF/emtricitabine[FTC]/rilpivirine[RPV]) or remain on their current TDF-based regimen (TDF/FTC/RPV). We hypothesized that the switch to TAF-based ART would result in a reduction in bone turnover at the skeletal sites of interest as measured by [^18^F]NaF-PET/CT.

## Materials and methods

### Study design and participants

PETRAM is a single-site, open-label, randomized controlled trial conducted at a large urban sexual health clinic in London, UK. The PETRAM population were males aged 40–65 years living with HIV-1, on a daily ART regimen of TDF/FTC/RPV for at least 6 months, virologically suppressed (<50 copies/ml) for at least 24 weeks, and with no known history of osteoporosis (T-score above -2.5 at the lumbar spine, femoral neck, or total hip when measured by DXA). Exclusion criteria included contraindication to the receipt of TAF or [^18^F]NaF-PET/CT scanning, anticipated additional imaging resulting in a cumulative total of ionizing radiation exceeding 50 millisieverts during the study period, exposure to treatments with bone metabolism effects within 12 months before recruitment (e.g. anabolic steroids, bisphosphonates, glucocorticoids for ≥3 months equivalent to prednisolone ≥5 mg daily), and active hepatitis C co-infection (positive antigen/PCR) within the preceding 24 weeks. Randomized individuals who were found to be incidentally osteoporotic on baseline DXA were withdrawn.

All participants provided written informed consent prior to study procedures. Ethical approval was authorized by the London – Riverside Research Ethics Committee (17/LO/2018) and the UK Administration of Radioactive Substances Advisory Committee. The study was registered at ClinicalTrials.gov (NCT#:03405012) and with EudraCT (2017–000677–36).

## Study procedures

### Randomization and masking

Eligible participants were randomized in a 1 : 1 fashion to remain on daily oral TDF/FTC/RPV (245/200/25 mg) or switch to TAF/FTC/RPV (25/200/25 mg), both as fixed-dose combination single tablets. Randomization was performed using a centralized computer-generated system. The randomization list was generated by a computer algorithm that ensured a maximum imbalance of three patients between groups at any point on the list. The list was encrypted and accessed by a program that revealed the next randomization as patients were entered into the trial. There was no stratification due to a small sample size target. The study was open-label, although the radiology departments were blinded to treatment allocation.

### Procedures

Eligible participants were assessed at randomization with planned follow-up visits at 24 and 48 weeks. Baseline scans were performed after randomization, but before the switch to TAF. Routine pathology (biochemistry, hematology, and HIV viral load), and stored serum and plasma (drawn fasted) for subsequent BTMs (e.g., cross-linked C telopeptides of Type I [CTX] and procollagen Type I N terminal propeptide [P1NP]), clinical assessment, and a 7-day recall for adherence assessment were performed at each study visit.

Stored samples were stored at -70^o^C and analyzed in the same batch at the end of the study. Plasma total P1NP (trimeric and the monomeric fractions) and CTX were measured by electrochemiluminescence immunoassay (ECLIA, Roche Diagnostics, Mannheim, Germany) following manufacturer's instructions at the Bioanalytical Facility, at the University of East Anglia. Typical laboratory performance of the assays is coefficient of variation less than 6.8% and CV less than 2.3% for CTX and P1NP, respectively.

Twenty-minute static PET/CT scans of the lumbar spine and hip were performed 1 h after injection of 90 MBq of [^18^F]NaF. A pre-PET CT scan provided attenuation correction of the PET images, and defined placement of the regions of interest for the PET scan analysis. A DXA scan measuring BMD at the lumbar spine, nondominant hip, and whole body was performed at the same three timepoints as the [^18^F]NaF-PET/CT (where possible). All [^18^F]NaF-PET/CTs were performed on the same scanner at King's College London and Guy's and St. Thomas’ PET Centre, all DXA scans were performed on the same DXA scanner at the Osteoporosis unit at Guy's and St Thomas’ NHS Foundation Trust. All scans were analyzed by the same two experienced PET scientists blinded to the group participants were randomized to. The detailed radiology procedures can be found in the supplementary (see Text, Supplemental Digital Content 1, Radiology Methods).

At the end of the study, all participants were transitioned back to TDF/FTC/RPV unless there was a clinical indication for them to remain on TAF in accordance with local clinical practices.

Due to the emergence of the Severe Acute Respiratory Syndrome CoronaVirus-2 (SARS-CoV-2) pandemic, subsequently named COVID-19, there was unavoidable disruption of study procedures from March to July 2020 in order to comply with public health lockdowns and minimization of in-person healthcare visits. The mitigation strategies during COVID-19 were described in a protocol amendment (June 2020, and a further protocol amendment in May 2021), and all participants were re-consented for the anticipated extension to the study and ART duration. ART supply was maintained for all participants, and scheduled visits continued virtually by telephone to assess for adverse events and adherence, with blood draws deferred as needed. For situations where scans were delayed, a further in-person visit was performed for sample collection. Individuals with delayed baseline scans who were allocated to switch remained on TDF/FTC/RPV until the baseline scans had occurred.

### Primary and secondary outcomes

The primary outcome was a change in regional bone formation at the hip and lumbar spine, measured using the SUV_mean_ from the [^18^F]NaF-PET/CT, between the baseline scan and the last scan. The secondary outcome was a change between baseline and the intermediate scan. There were a number of exploratory outcomes including changes in spine and hip BMD and in BTMs; some of the exploratory outcomes (as per protocol version 4.0 09-May-2021) presented in this manuscript are detailed in the supplementary (see Text, Supplemental Digital Content 2, Exploratory Outcomes).

### Statistical analysis

The planned sample size was 30 participants (15 per group). This was estimated to give 90% power to detect a significant difference between groups (at 5% significance), assuming a 25% difference in the primary outcome measures (measured as percentage-change from baseline) and a within-group standard deviation of 20%.

The primary analysis compared the two groups as allocated (intention to treat, ITT). The high levels of adherence to trial regimens (see Results) obviated the need for an on-treatment analysis. Data were analyzed using analysis of covariance adjusting for baseline value and the interval between the baseline measurement and the final measure. All variables were analyzed on a log_10_ scale; results were back-transformed to the original scale and expressed as the predicted (an estimation based for each group as a whole derived from the individual participant data that adjusts for the variable time interval) percentage change from first measurement to 48 weeks. Data were represented graphically as scatterplots (comparing the last or intermediate scan versus baseline) and as longitudinal plots of individual trajectories. All analyses were performed in STATA version 15 (where STATA is a complete, integrated statistical software package developed by StataCorp) [23].

In the analysis of the BTM data, if there was discordance for some participants between the timing of the blood draw for BTM and their [^18^F]NaF-PET/CT, the blood draw was repeated prior to the delayed scans.

### Role of funding source

University College London was the trial sponsor. Gilead Sciences funded the study as an Investigator Sponsored Research/Collaborative Study project (IN-UK-366–4216 27) and provided the study supply of TAF/FTC/RPV. The UK National Health System provided the TDF/FTC RPV fixed-dose combination tablets. Gilead Sciences played no role in the collection, analysis, or interpretation of the study data; they reviewed the final manuscript prior to submission.

## Results

**Fig. 1:**
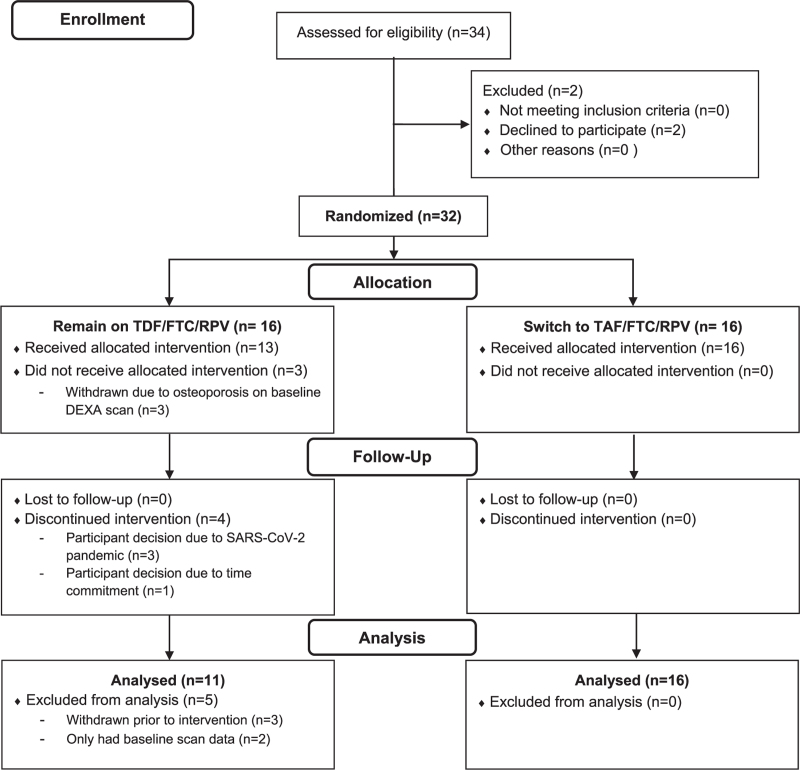
CONSORT diagram.

Thirty-two participants (16 TAF:16 TDF) were randomized between 31 August 2018 and 09 March 2020 (Fig. 1). Three participants (all assigned to remain on TDF) were found to have osteoporosis on their baseline scan and were excluded. Baseline characteristics are summarized in Table 1. Mean age (SD) was 51 (5.2) years, 76% (*n* = 22) white ethnicity, BMI 25.5 (2.9) kg/m^2^; mean CD4^+^ T-cell count was 529 (155) cells/μl. Fifty-five percent (*n* = 16) were vitamin D sufficient, 38% (*n* = 11) insufficient, and 7% (*n* = 2) deficient. Characteristics were reasonably balanced between the groups, apart from a longer duration of HIV in the TDF group (mean 12.7 [5.8] years in TAF group versus 17.5 [7.8] years in TDF group) and prior duration of TDF/FTC/RPV use (mean 3.8 [1.8] years in TAF group versus 4.5 [1.3] years in TDF group).

**Table 1:** Baseline characteristics.

Characteristic	–	TAF (*n* = 16)	TDF (*n* = 13)	Total (*n* = 29)
White ethnicity	*n* (%)	12 (75)	10 (77)	22 (76)
Age (years)^a^	Mean (SD)	49.6 (5.3)	52.7 (4.8)	51.0 (5.2)
Time since HIV diagnosis (years)	Mean (SD)	12.7 (5.8)	17.5 (7.8)	14.9 (7.1)
CD4^+^ T-cell count (cells/μl)	Mean (SD)	525 (162)	534 (152)	529 (155)
eGFR (ml/min/1.73 m^2^)	Mean (SD)	112.9 (22.6)	103.9 (22.1)	108.9 (22.4)
Duration of TDF/FTC/RPV use (months)	Mean (SD)	45 (22)	54 (16)	49 (20)
Weight (kg)	Mean (SD)	84.3 (12.9)	82.6 (6.2)	83.5 (10.3)
BMI (kg/m^2^)	Mean (SD)	25.8 (3.2)	25.1 (2.7)	25.5 (2.9)
FRAX score^b^	Mean (SD)	3.4 (1.4)	3.1 (0.6)	3.3 (1.1)
T-score Femoral Neck	Mean (SD)	−0.8 (1.2)	−0.7 (0.8)	−0.8 (1.0)
Vitamin D	–	–	–	–
Sufficient (>50 nmol/l)	*n* (%)	8 (50)	8 (61)	16 (55)
Insufficient (25–50 nmol/l)	*n* (%)	7 (44)	4 (31)	11 (38)
Deficient (<25 nmol/l)	*n* (%)	1 (6)	1 (8)	2 (7)
Smoking status	–	–	–	–
Current	*n* (%)	5 (31)	3 (23)	8 (28)
Past	*n* (%)	6 (38)	4 (31)	10 (34)

*n* = 2 of vitamin D deficient and *n* = 7 of insufficient started vitamin D supplementation.

a All participants are men, aged 40–65 years by inclusion criteria. FRAX, Fracture risk assessment tool; FTC, emtricitabine; RPV, rilpivirine; SD, standard deviation; TAF, tenofovir alafenamide fumarate; TDF, tenofovir disoproxil fumarate.

b Ten-year probability of major osteoporotic fracture (%).

All participants had an uninterrupted supply of trial medication throughout follow-up. Sixteen participants reported never missing doses; the remaining nine reported missing at most one dose in the previous seven days. The high level of reported adherence was supported by HIV viral load measurements. Only one participant experienced a transient viral load blip (123 copies/ml) at week 63, with subsequent measures of less than 50 copies/ml. There was one serious adverse event (TAF), a case of prostate cancer diagnosed at 42 weeks deemed unrelated to trial medication, and three participants (1 TAF:2 TDF) had a grade 3 or 4 clinical adverse event considered as unrelated to study drugs.

Nineteen participants had two follow-up scans, in accordance with the study protocol, while eight had a single follow-up scan only. Three participants (all TDF) had no follow-up scans and did not contribute to further analyses. The first follow-up scan was performed at a median of 28 weeks postbaseline (interquartile range [IQR] 25–36 weeks, range 23–87 weeks); the final scan was performed at a median of 55 weeks postbaseline (IQR 49–71 weeks, range 23–103 weeks, Figure 1S [see Figure, Supplemental Digital Content 4, comparison of original study and implemented study timeline]).

**Fig. 2:**
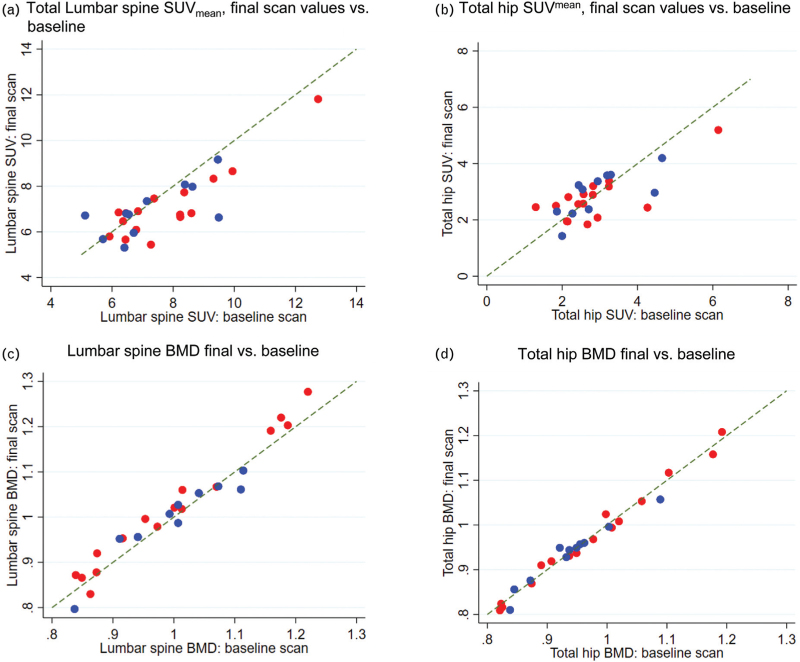
(a-d) Final versus baseline plots of standardized uptake values measured using [^18^F]NaF-PET/CT and bone mineral density using dual x-ray absorptiometry scans.

**Fig. 3:**
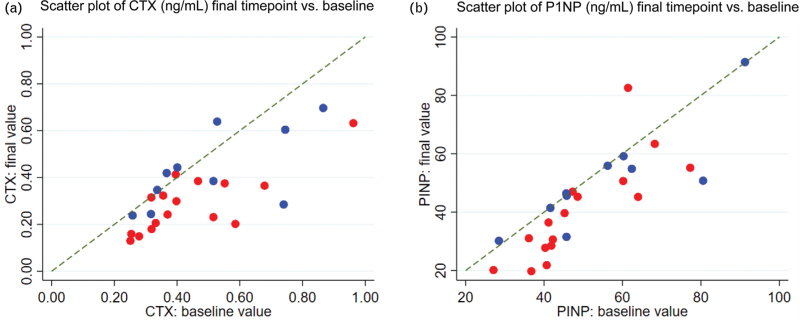
(a,b) Plots of bone turnover markers measured at final assessment versus baseline assessment.

Figure 2a,b shows the association between the SUV_mean_ measured at the final and baseline [^18^F]NaF-PET/CT scans for lumbar spine and total hip (primary outcomes). Table 2 summarizes the results of the analysis of covariance, including predicted changes at 48 weeks. There was a systemic decrease in lumbar spine SUV_mean_ for both groups (predicted -7.9% [95% confidence interval, 95% CI -14.4, -1.5] change for TAF at last scan versus -5.3% [-12.1, 1.5] change for TDF), but the difference between the groups was not statistically significant (*P* = 0.57, Table 2). For both groups, there was no change in total hip SUV_mean_ (+0.3% [-12.2, 12.8] TAF versus +2.9% [-11.1, 16.9] TDF), again with no evidence of a difference between the groups (*P* = 0.77). The absolute and percentage changes for the two primary outcomes are described in the supplementary (see Figures, Supplemental Digital Content 5, lumbar spine SUV, Figures 2Sb-c; Supplemental Digital Content 6, TH SUV, Figures 3Sb-c).

(a) Total Lumbar spine SUV_mean_, final scan values versus baseline. (b) Total hip SUV_mean_, final scan values versus baseline. (c) Lumbar spine BMD final versus baseline. (d) Total hip BMD final versus baseline.

**Table 2:** Analysis of covariance for bone turnover, bone mineral density, and bone turnover markers: baseline to final assessments.

	Baseline mean (SD)		Predicted relative change (%) at 48 weeks (95% CI)^a^			
Parameter	TAF	TDF	TAF	TDF	Relative difference (%): TAF versus TDF (95% CI)	*P*
[^18^F]NaF-PET/CT SUV^b^						
Lumbar spine	7.81 (1.77)	7.27 (1.49)	−7.9 (−14.4, −1.5)	−5.3 (−12.1, 1.5)	−2.8 (−12.0, 6.5)	0.57
Total hip	2.83 (1.11)	2.94 (0.92)	+0.3 (−12.2, 12.8)	+2.9 (−11.1, 16.9)	−2.6 (−19.4, 14.3)	0.77
DXA BMD (g/cm^2^)						
Lumbar spine	0.999 (0.130)	0.998 (0.086)	+1.7 (0.3, 3.1)	−0.3 (−1.8, 1.2)	+2.0 (0.0, 4.0)	0.06
Total hip	0.972 (0.118)	0.937 (0.072)	−0.2 (−1.1, 0.7)	−0.2 (−1.2, 0.8)	0.0 (−1.3, 1.4)	0.94
BTM (ng/ml)						
CTX	0.440 (0.187)	0.508 (0.210)	−35.3 (−45.7, −24.9)	−11.6 (−28.8, 5.6)	−26.8 (−44.7, −8.9)	0.02
P1NP	48.6 (13.6)	55.8 (18.8)	−17.6 (−26.6, −8.5)	−6.9 (−19.2, 5.4)	−11.4 (−26.2, 3.3)	0.17

BMD, bone mineral density; BTM, bone turnover marker; CI, confidence interval; CT, computerized tomography; CTX, cross-linked C telopeptides of Type I; DXA, dual-energy x-ray absorptiometry; P1NP, procollagen Type 1 N terminal propeptide; SD, standard deviation; SUV, standardized uptake value; TAF, tenofovir alafenamide fumarate; TDF, tenofovir disoproxil fumarate.

a Prediction according to overall mean baseline value.

b Unitless.

Lumbar spine and total hip BMD at final and baseline scans were highly correlated (*r* = 0.97 and *r* = 0.99, respectively), attesting to the high reproducibility of this method (Fig. 2c,d, respectively). In the TAF group, there was a nonsignificant trend toward improvement in lumbar spine BMD compared to TDF (predicted increase compared to TDF of 2.0% [95% CI: 0.0-- 4.0, *P* = 0.06], Table 2, Figure 4Sb-c [see Figure, Supplemental Digital Content 7, lumbar spine BMD changes]). total hip BMD showed no overall change in either group (Table 2, Figure 5Sb-c [see Figure, Supplemental Digital Content 8, total hip BMD changes]). The BTMs, CTX and P1NP, decreased markedly in the TAF group, with predicted reductions at last scan of -35.3% (-45.7, -24.9) and -17.6% (-26.6, -8.5), respectively (Table 2, Fig. 3a,b). This change occurred early for both CTX and P1NP, with no significant further change between this and the final measurement (Table 1S [See Table, Supplemental Digital Content 3, analysis intermediate scans]; Figures 6Sa-c and 7Sa-c [see Figure, Supplemental Digital Content 9 and 10, CTX and P1NP changes, respectively]). Smaller decreases were observed in the TDF group: -11.6% (-28.8, 5.6) for CTX and -6.9% (-19.2, 5.4) for P1NP. The difference between the arms was statistically significant for changes in CTX (*P* = 0.02) but not for P1NP (*P* = 0.17, Table 2 and Fig. 3).

(a) Scatter plot of CTX (ng/ml) final timepoint versus baseline. (b) Scatter plot of P1NP (ng/ml) final timepoint versus baseline.

All the above analyses were repeated using the baseline to intermediate scans/visits (See Table, Supplemental Digital Content 3, analysis intermediate scans and Figures 2Sa-7Sa, Supplemental Digital Content 5–10, baseline-intermediate measurements SUV_mean_, BMD, CTX, and P1NP). Note that these analyses are not independent of each other, as the eight participants with a single follow-up scan contribute the same data point to both analyses. These analyses gave broadly similar conclusions, including the effect of switching from TDF to TAF on BTMs (Table 1S [See Table, Supplemental Digital Content 3, analysis intermediate scans]; Figures 6Sa-c and 7Sa-c [see Figure, Supplemental Digital Content 9 and 10, CTX and P1NP changes respectively]), Figure 4Sa-c [see Figure, Supplemental Digital Content 7, lumbar spine BMD changes]). For reference, the protocol is included in Supplemental Digital Content 11.

## Discussion

While this study observed a generalized decrease in the lumbar spine SUV_mean_ and no change in the total hip SUV_mean_, as measured by [^18^F]NaF-PET/CT for both TDF and TAF, there was no significant difference between the two prodrugs of tenofovir over a median of 55 weeks of follow-up. There was a trend toward an increase in lumbar spine BMD in the TAF group that failed to reach the level of significance, not mirrored at the hip for either arm. Importantly, there were no convincing beneficial effects of TAF compared to TDF on bone turnover detected on the radiological platforms used in PETRAM. However, of the plasma bone turnover markers, there was a significant reduction in CTX for the TAF participants compared to TDF, with a smaller decrease in P1NP that was similar for both groups. These biochemical changes largely occurred early, between baseline and participants’ first follow-up scans (median 28 weeks, IQR 25–36), and then stabilized.

When considered together, these results suggest that this group of virologically suppressed participants with normal CD4^+^ T-cell counts are in a state of increased bone turnover, which was reduced by the switch to TAF. CTX is a bone resorption marker and P1NP a bone formation marker, so when participants switched to TAF, the CTX quickly declines, followed by the P1NP as the rate of bone turnover decreases. The normalization of bone turnover yields a marginal increase in the lumbar spine BMD for the TAF group, as the bone lost in the high remodeling state is restored. This is not observed with the hip, likely because bone turnover changes occur predominantly in trabecular bone. Similarly, the decrease in lumbar SUV_mean_ is probably reflective of the decrease in bone turnover, which would again be less marked in the hip. The lack of difference between groups may be a consequence of the study's small sample size. Overall, these findings are similar to what is observed when a patient with osteoporosis is treated with a weak bisphosphonate [24].

This study is the first utilization of a novel radiological platform to assess bone turnover in a cohort living with HIV. Emtricitabine and rilpivirine were purposefully used as the additional components of the ART regimen in both groups, allowing for any possible ART effect to be attributable to the different tenofovir prodrugs used. The study is also unique in being able to amalgamate information extracted from clinically utilized DXA BMD, the novel SUV_mean_ data, and the bone turnover markers to formulate a better understanding of the process underlying the reported observations of bone loss with TDF [25,26], and the benefits of switching to TAF [8].

The study was limited by a number of factors, including the very small sample size, as well as more participant withdrawal in the TDF group. In addition, we deliberately excluded cis-women to avoid confounding by menopausal hormone changes. Hence, our findings cannot be extrapolated to cis-women living with HIV. In addition, the unexpected disruption caused by the COVID-19 pandemic generated discordance in the scheduling of the scans and duration of participation in the study and necessitated an amendment to both the timing of the primary and secondary outcomes, as the originally planned time points of 24 and 48 weeks were impossible to adhere to. Finally, we did not control for vitamin D use in participants as part of the study design beyond simple medication review, although all participants found to be insufficient or deficient at baseline were supplemented as per UK guidelines [27].

Contrary to our hypothesis, TAF did not have a significant impact on BMD or SUV_mean_ compared to TDF, even with the longer drug exposures than originally planned due to delayed scans during the COVID-19 pandemic. However, the combination of a rapidly decreasing CTX, a trend toward (albeit nonsignificant) increased lumbar spine BMD, and a decrease in lumbar SUV_mean_, signals a possible transition from a high to a more normalized bone turnover state with TAF. These CTX and BMD changes have also been observed in other studies [28]. This sheds further light on the potential mechanisms of bone loss with TDF and should help guide the design of future studies, particularly ones involving switching from TDF-based regimens to two-drug combinations.

## Acknowledgements

The authors wish to thank all the participants for their participation, especially during the challenges faced by many during the first year especially of the COVID-19 pandemic.

PETRAM was funded by an independent academic grant IN-UK-366–4216 27 in support of investigator-initiated research from Gilead Sciences. Pett and Dunn receive funding in support of their salary from the Medical Research Council, United Kingdom (grant MC_UU_00004/03 and MC_UU_00004/04). Pool is funded by an NIHR Academic Clinical Fellowship. The authors also acknowledge financial support from the Wellcome/Engineering and Physical Sciences Research Council Centre for Medical Engineering at King's College London (WT 203148/Z/16/Z) and National Institute for Health Research Biomedical Research Centre at Guy's & St Thomas’ Hospitals and King's College London.

All authors had full access to all data and accept responsibility to submit for publication. S.L.P., A.E.B.M., A.M., D.D., R.G., A.T. designed the study. E.R.M.P., J.E.B., S.L.P., A.A.P., A.B., D.S., J.S. recruited and managed the participants according to the protocol. D.D. performed the statistical analysis, and this was verified by J.E.B. J.E.B., D.D., S.L.P., and A.E.B.M. wrote the first draft of the manuscript. A.E.B.M., G.M.B., G.K., G.J.R.C., C.R., A.H., J.J., and D.S.D. conducted the radiological assessments and analysis. All authors reviewed the draft manuscript prior to submission.

Data will be shared according to the IGH's controlled access approach, based on the following principles: No data should be released that would compromise an ongoing trial or study.

There must be a strong scientific or other legitimate rationale for the data to be used for the requested purpose.

Investigators who have invested time and effort into developing a trial or study should have a period of exclusivity in which to pursue their aims with the data before key trial data are made available to other researchers.

The resources required to process requests should not be underestimated, particularly successful requests which lead to preparing data for release. Therefore, adequate resources must be available in order to comply in a timely manner or at all, and the scientific aims of the study must justify the use of such resources.

Data exchange complies with Information Governance and Data Security Policies in all the relevant countries.

Data will be available for sharing from 2023 onwards. Researchers wishing to access PETRAM data should contact the Trial Management Group in the first instance.

### Conflicts of interest

There are no conflicts of interest.

## Supplementary Material

Supplemental Digital Content

## Supplementary Material

Supplemental Digital Content

## Supplementary Material

Supplemental Digital Content

## Supplementary Material

Supplemental Digital Content

## Supplementary Material

Supplemental Digital Content

## Supplementary Material

Supplemental Digital Content

## Supplementary Material

Supplemental Digital Content

## Supplementary Material

Supplemental Digital Content

## Supplementary Material

Supplemental Digital Content

## Supplementary Material

Supplemental Digital Content

## Supplementary Material

Supplemental Digital Content
