# Supplementary material for: Bone turnover change after randomized switch from tenofovir disoproxil to tenofovir alafenamide fumarate in men with HIV
Source: AIDS. 2024 Feb 1;38(4):521–9. doi: 10.1097/QAD.0000000000003811 (PMC10906193; doi:10.1097/QAD.0000000000003811)
Supplement: Supplemental Digital Content [file aids-38-521-s001.docx]

**Supplemental Digital Content 1**

**Text - Radiology methods**

**[^18^F]NaF-PET/CT**

*Image acquisition*

Following an intravenous injection of 84 ± 11 MBq (mean ± standard deviation) [^18^F]NaF, a 20-minute static scan (5 min per bed position) of the lumbar spine and both hips was acquired starting one hour after injection on a GE Discovery 710 PET/CT scanner (General Electric Medical Systems, Waukesha, WI, USA) at baseline and at follow-up visits 24 and 48 weeks later. When planned follow-up visits were disrupted by lockdowns due to the COVID-19 pandemic, they were rescheduled as soon as possible afterwards. Subjects were positioned supine and a low-dose CT (140 kVp, 20 milliampere seconds (mAs)) image was acquired for attenuation correction and image segmentation prior to the PET scan.

*Image reconstruction*

The PET emission data were acquired in list mode, and the CT data were used to correct for attenuation. The data were then reconstructed with a 3D ordered subset expectation maximisation (3D-OSEM) algorithm with 2 iterations and 24 subsets, and a 6.4 mm post-reconstruction Gaussian filter was applied. The images had a reconstruction matrix size of 244 x 244 and a 2.73 x 2.73 x 3.27 mm^3^ voxel size. All CT images were reconstructed in a 512 x 512 matrix and a 0.98 x 0.98 x 2.50 mm^3^ voxel size.

*Image analysis*

All activity measurements were corrected for radioactive decay back to the time of injection. The PET scanner was recalibrated at 3 monthly intervals throughout the study. For the PET analysis, the bone regions of interest (ROIs) used for the PET scan analyses were segmented using the CT scan images. For the analysis of the lumbar spine, an elliptical ROI was placed in the middle of each vertebra from L1 to L4, excluding the end plates on the corresponding CT segmented scan. The PET and CT images were assumed to already be aligned with any patient movement picked up by eye and no additional registration was used. The regions were projected on the PET scans in order to calculate the mean activity concentration (kBq/ml) in each region. The mean SUV (SUV_mean_) for each vertebra was calculated using the equation:

$$SUV=\frac{Tracer Uptake Within ROI\left( units:{kBq}/{ml} \right)*Body Weight\left( units:g \right)}{Injected Activity\left( units:kBq \right)}$$

The final lumbar spine SUV was based on the average of the four individual vertebrae. For the analysis of the total hip, a region of mixed trabecular and cortical bone was drawn between the femoral neck and the lesser trochanter, anatomically equivalent to the total hip ROI used in DXA scanning.

**DXA**

DXA scans were performed at the lumbar spine (L1-L4) (LS) and the non-dominant hip, including the total hip (TH) site, using a Hologic Discovery (Hologic, Marlborough, MA, USA) at baseline, 24 and 48 weeks. All scans were performed on the same machine. The majority of DXA scans were performed on the same day as the PET scans, though this was not always possible due to COVID-19 restrictions. Areal BMD (g/cm^2^) was measured at the LS and TH.
