# Supplementary material for: Bone turnover change after randomized switch from tenofovir disoproxil to tenofovir alafenamide fumarate in men with HIV
Source: AIDS. 2024 Feb 1;38(4):521–9. doi: 10.1097/QAD.0000000000003811 (PMC10906193; doi:10.1097/QAD.0000000000003811)
Supplement: Supplemental Digital Content [file aids-38-521-s002.docx]

**Supplemental Digital Content 2**

**TEXT - Exploratory outcomes**

1. To compare the changes in bone biomarkers with changes in [^18^F]NaF-PET/CT between baseline, mid-point, and last study scans.
2. To compare changes in bone mineral density at the lumbar spine as measured by DXA between baseline, mid-point, and last study scans.
