# Supplementary material for: Bone turnover change after randomized switch from tenofovir disoproxil to tenofovir alafenamide fumarate in men with HIV
Source: AIDS. 2024 Feb 1;38(4):521–9. doi: 10.1097/QAD.0000000000003811 (PMC10906193; doi:10.1097/QAD.0000000000003811)
Supplement: Supplemental Digital Content [file aids-38-521-s003.docx]

**Supplemental Digital Content 3**

Table 1S. Analysis of bone turnover, bone mineral density, and bone turnover markers: intermediate assessments

| **Parameter** | **Baseline mean (SD)** | | **Predicted relative change (%)**  **at 24 weeks (95 % CI)*** | | **Relative difference (%): TAF vs. TDF (95% CI)** | **p-value** |
| --- | --- | --- | --- | --- | --- | --- |
|  | **TAF** | **TDF** | **TAF** | **TDF** |  |  |
| [^18^F]NaF-PET/CT SUV_mean_^†^ |  | | | | | |
| Lumbar spine | 7.81 (1.77) | 7.27 (1.49) | -5.8 (-10.7, -0.8) | -4.9 (-10.3, 0.5) | -0.9 (-8.0, 6.2) | 0.80 |
| Total hip | 2.83 (1.11) | 2.94 (0.92) | +11.4 (-4.6, 27.3) | +5.7 (-10.3, 21.7) | +5.4 (-15.0, 25.7) | 0.60 |
| DXA BMD (g/cm^2^) |  | | | | | |
| Lumbar spine | 0.999 (0.130) | 0.998 (0.086) | +1.9 (0.2, 3.7) | -0.3 (-2.1, 1.5) | +2.3 (0.1, 4.4) | 0.05 |
| Total hip | 0.972 (0.118) | 0.937 (0.072) | +0.1 (-0.6, 0.9) | +0.4 (-0.4, 1.2) | -0.3 (-1.2, 0.7) | 0.56 |
| BTM (ng/mL) |  | | | | | |
| CTX | 0.440 (0.187) | 0.508 (0.210) | -31.2 (-41.9, -20.4) | -6.1 (-21.9, 9.7) | -26.7 (-42.4, -11.0) | 0.009 |
| PINP | 48.6 (13.6) | 55.8 (18.8) | -18.2 (-31.0, -5.4) | -9.5 (-24.6, 5.7) | -9.6 (-29.0, 9.8) | 0.37 |

*Prediction according to overall mean baseline value. ^†^Unitless

BMD: bone mineral density; BTM: bone turnover marker; CI: confidence interval; CT: computerised tomography; CTX: cross-linked C telopeptides of Type I; DXA: dual-energy x-ray absorptiometry; PET: positron emission tomography; P1NP: procollagen Type I N terminal propeptide; SD: standard deviation; SUV: standardised uptake value; TAF: tenofovir alafenamide fumarate; TDF: tenofovir disoproxil fumarate
