# Supplementary material for: Bone turnover change after randomized switch from tenofovir disoproxil to tenofovir alafenamide fumarate in men with HIV
Source: AIDS. 2024 Feb 1;38(4):521–9. doi: 10.1097/QAD.0000000000003811 (PMC10906193; doi:10.1097/QAD.0000000000003811)
Supplement: Supplemental Digital Content [file aids-38-521-s004.pptx]

## Slide 1
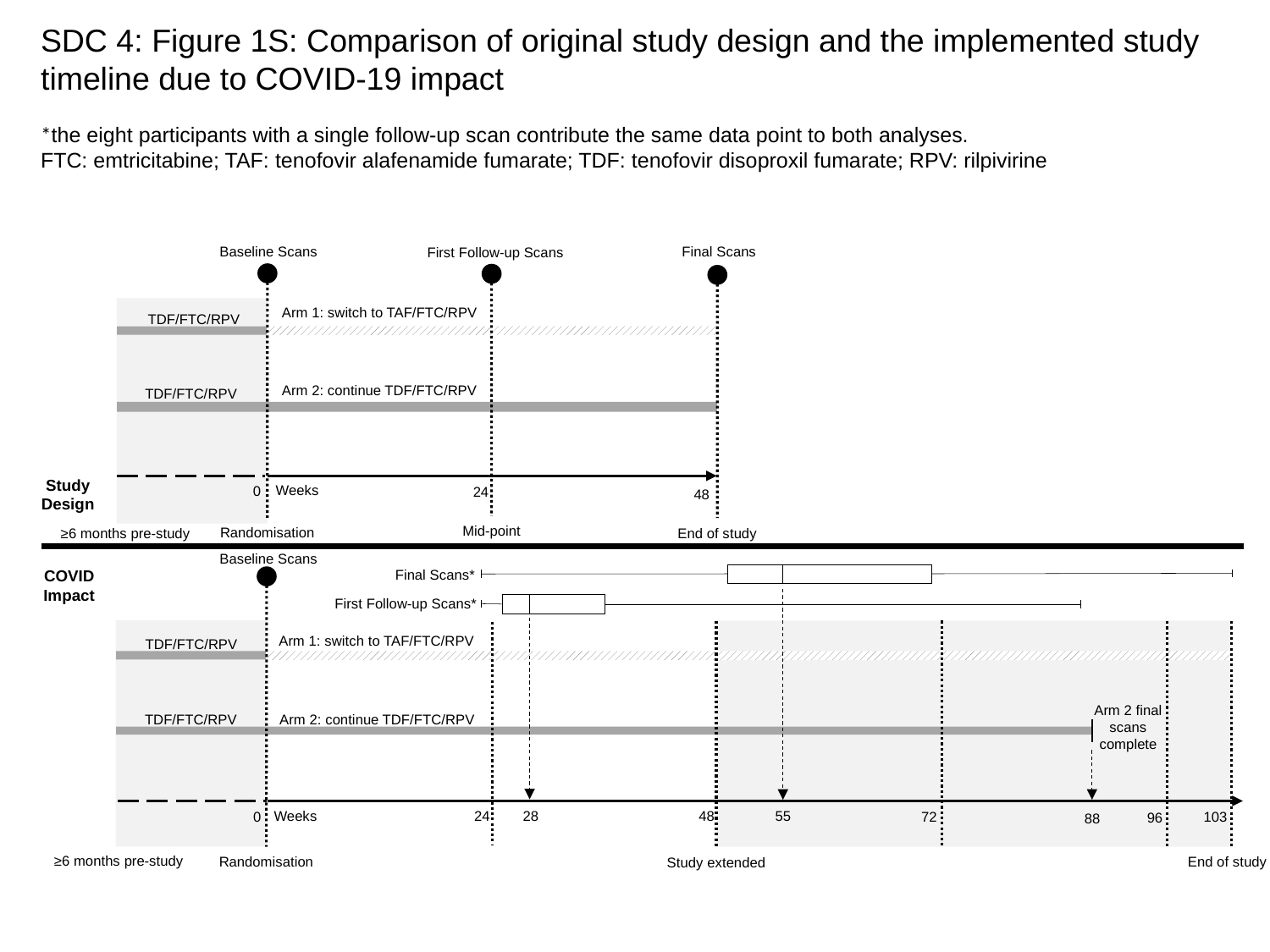

SDC 4: Figure 1S: Comparison of original study design and the implemented study timeline due to COVID-19 impact
*the eight participants with a single follow-up scan contribute the same data point to both analyses.
FTC: emtricitabine; TAF: tenofovir alafenamide fumarate; TDF: tenofovir disoproxil fumarate; RPV: rilpivirine
Baseline Scans
Final Scans
First Follow-up Scans
Arm 1: switch to TAF/FTC/RPV
TDF/FTC/RPV
Arm 2: continue TDF/FTC/RPV
TDF/FTC/RPV
Study Design
Weeks
0
24
48
Mid-point
Randomisation
End of study
≥6 months pre-study
Baseline Scans
Final Scans*
COVID Impact
First Follow-up Scans*
Arm 1: switch to TAF/FTC/RPV
TDF/FTC/RPV
Arm 2 final scans complete
TDF/FTC/RPV
Arm 2: continue TDF/FTC/RPV
24
Weeks
28
48
55
0
72
103
96
88
≥6 months pre-study
Randomisation
End of study
Study extended
