# Supplementary material for: Bone turnover change after randomized switch from tenofovir disoproxil to tenofovir alafenamide fumarate in men with HIV
Source: AIDS. 2024 Feb 1;38(4):521–9. doi: 10.1097/QAD.0000000000003811 (PMC10906193; doi:10.1097/QAD.0000000000003811)
Supplement: Supplemental Digital Content [file aids-38-521-s008.pptx]

## Slide 1
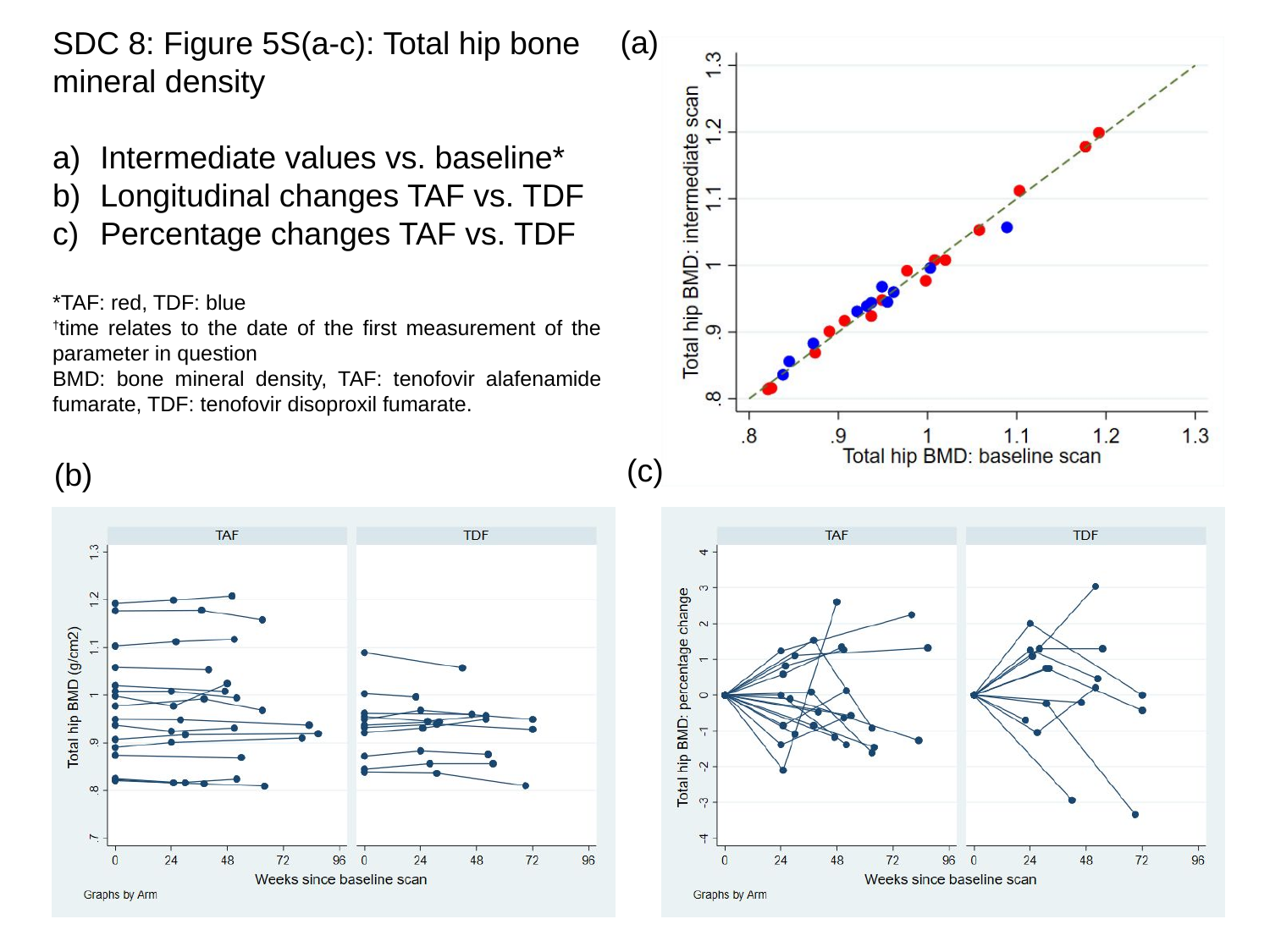

(a)
SDC 8: Figure 5S(a-c): Total hip bone mineral density
Intermediate values vs. baseline*
Longitudinal changes TAF vs. TDF
Percentage changes TAF vs. TDF
*TAF: red, TDF: blue
†time relates to the date of the first measurement of the parameter in question
BMD: bone mineral density, TAF: tenofovir alafenamide fumarate, TDF: tenofovir disoproxil fumarate.
(c)
(b)
