# Supplementary material for: Bone turnover change after randomized switch from tenofovir disoproxil to tenofovir alafenamide fumarate in men with HIV
Source: AIDS. 2024 Feb 1;38(4):521–9. doi: 10.1097/QAD.0000000000003811 (PMC10906193; doi:10.1097/QAD.0000000000003811)
Supplement: Supplemental Digital Content [file aids-38-521-s010.pptx]

## Slide 1
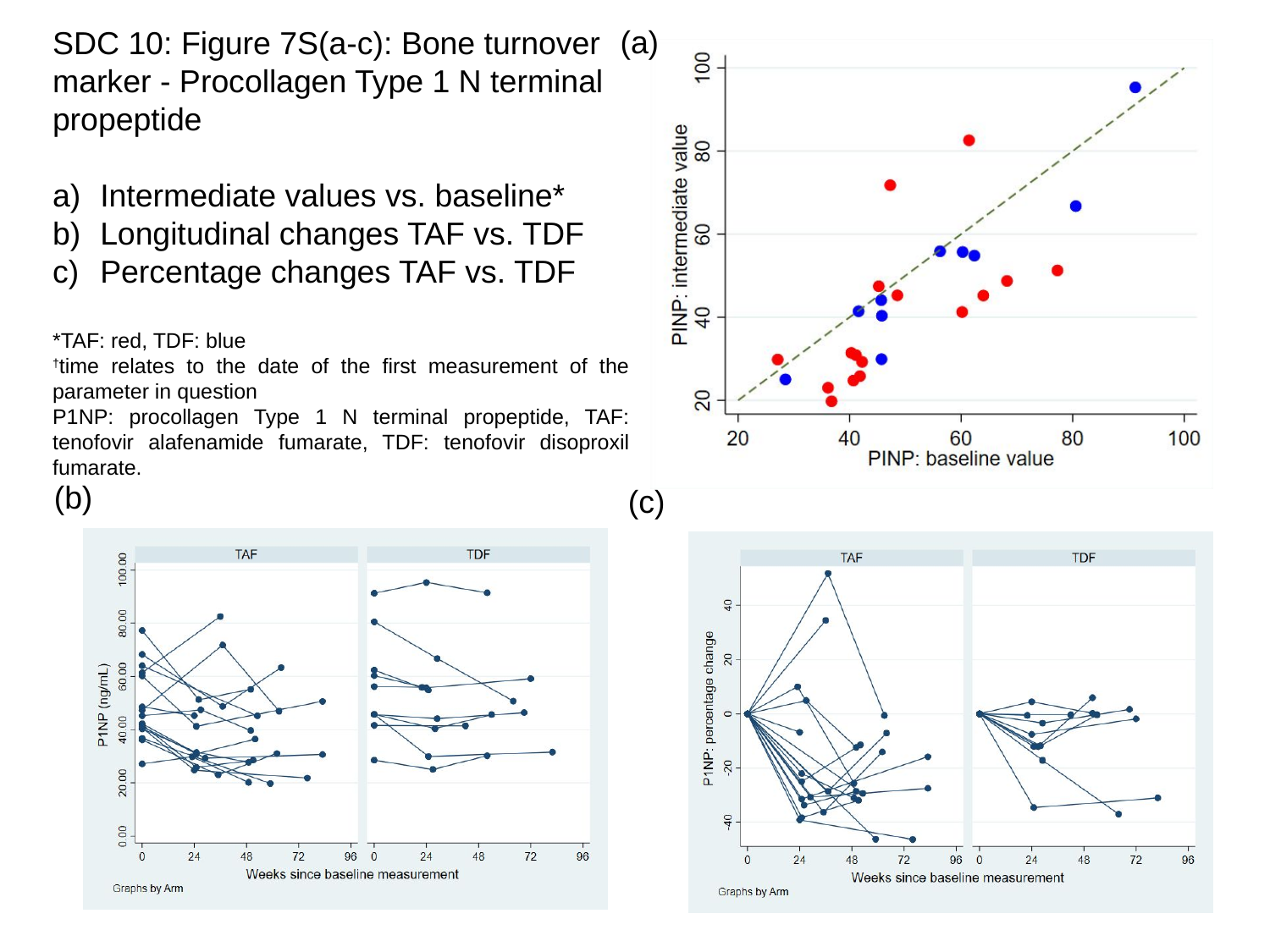

(a)
SDC 10: Figure 7S(a-c): Bone turnover marker - Procollagen Type 1 N terminal propeptide
Intermediate values vs. baseline*
Longitudinal changes TAF vs. TDF
Percentage changes TAF vs. TDF
*TAF: red, TDF: blue
†time relates to the date of the first measurement of the parameter in question
P1NP: procollagen Type 1 N terminal propeptide, TAF: tenofovir alafenamide fumarate, TDF: tenofovir disoproxil fumarate.
(b)
(c)
