# Supplementary material for: Bone turnover change after randomized switch from tenofovir disoproxil to tenofovir alafenamide fumarate in men with HIV
Source: AIDS. 2024 Feb 1;38(4):521–9. doi: 10.1097/QAD.0000000000003811 (PMC10906193; doi:10.1097/QAD.0000000000003811)
Supplement: Supplemental Digital Content [file aids-38-521-s011.docx]

**
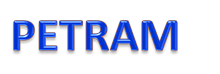
**

**Understanding changes in treatment-related regional bone turnover using 18F-fluoride-PET/CT in HIV-1-infected men: PETRAM study**

Version and date of protocol: 09 May 2021, Version 4.0

Sponsor: University College London (UCL)

Sponsor protocol number: 16/0657

EudraCT Number: 2017-000677-36

REC Reference Number: 17/LO/2018

Clinical Trials. gov: NCT03405012

| **Chief investigator:**  Dr Sarah Pett  UCL Centre for Sexual Health and HIV Research  The Mortimer Market Centre  off Capper Street; London, WC1B 6JB  Email: s.pett@ucl.ac.uk | **Sponsor Representative**:  Pushpsen Joshi,  Joint Research Office, UCL,  1st Floor Maple House,  149 Tottenham Court Road,  London W1T 7NF  Email: randd@uclh.nhs.uk  Postal address:  Joint Research Office, UCL  Gower Street,  London WC1E 6BT  Email: pushpsen.joshi1@nhs.net |
| --- | --- |

**Signatures**

The Chief Investigator and the JRO have discussed this protocol. The investigator agrees to perform the investigations and to abide by this protocol.

The investigator agrees to conduct the trial in compliance with the approved protocol, the UK Data Protection Act (1998), the Trust Information Governance Policy (or other local equivalent), the current Research Governance Framework, the Sponsor’s SOPs, and other regulatory requirements as amended.

| **Chief investigator** |  |  |
| --- | --- | --- |
| UCL | Signature | Date |
| **Sponsor**  Sponsor representative |  |  |
| UCL | Signature | Date |
|  |  |  |

**GENERAL INFORMATION**

This document describes the ‘Understanding changes in treatment-related regional bone turnover using 18F-fluoride-PET/CT (18F-PET/CT) in HIV-1-infected men: PETRAM study’, coordinated by the UCL Research Department of Infection and Population Health, and provides information about procedures for entering participants into it. Every care has been taken in drafting this protocol, but corrections or amendments may be necessary. Clinical problems relating to this study should be referred to the Chief Investigator.

**COMPLIANCE**

The study will be conducted in compliance with the approved protocol, the Declaration of Helsinki (fourth revision 1996) the principles of Good Clinical Practice (GCP), the UK Data Protection Act (DPA number Z5886415), the Commission Directive 2005/28/EC with the implementation in national legislation in the UK by Statutory Instrument 2004/1031 and subsequent amendments, and NHS research governance and other regulatory requirements, as appropriate in the participating centres.

**SPONSOR**

UCL is the Sponsor of this study. Queries relating to sponsorship of this study should be addressed via the study management team.

As a University organisation we use personally-identifiable information to conduct research to improve health, care and services. As a publicly-funded organisation, we have to ensure that it is in the public interest when we use personally-identifiable information from people who have agreed to take part in research. This means that when you agree to take part in a research study, we will use your data in the ways needed to conduct and analyse the research study. Your rights to access, change or move your information are limited, as we need to manage your information in specific ways in order for the research to be reliable and accurate. If you withdraw from the study, we will keep the information about you that we have already obtained. To safeguard your rights, we will use the minimum personally-identifiable information possible.

Health and care research should serve the public interest, which means that we have to demonstrate that our research serves the interests of society as a whole. We do this by following the UK Policy Framework for Health and Social Care Research.

If you wish to raise a complaint on how we have handled your personal data, you can contact our Data Protection Officer who will investigate the matter. If you are not satisfied with our response or believe we are processing your personal data in a way that is not lawful you can complain to the Information Commissioner’s Office (ICO).

Our Data Protection Officer is Lee Shailer and you can contact them at l.shailer@ucl.ac.uk.

**FUNDING**

An independent, academic grant in support of investigator initiated research from Gilead Sciences**.**

**STUDY REGISTRATION**

PETRAM study has been registered with EudraCT identified with the number 2017-000677-36.

SAE REPORTING

Following the standard PETRAM procedures.

Within 24 hours of becoming aware of an SAE, please email
the completed SAE form to the PI of the study  [sarah.pett@nhs.net](mailto:%20sarah.pett@nhs.net)

**study Administration**

Please direct all queries to the Chief Investigator.

**Coordinating Site**

| UCL Centre for Sexual Health and HIV Research  The Mortimer Market Centre  Capper Street, London, WC1B 6JB | Phone:  Fax:  Email: | 020 3108 2100  020 3108 2079  s.pett@ucl.ac.uk |
| --- | --- | --- |

**study management team**

| Chief Investigator | Dr. Sarah Pett | s.pett@ucl.ac.uk |
| --- | --- | --- |
| KCL PI and Co-Investigator | Dr. Amelia Moore | amelia.moore@kcl.ac.uk |
| Statistician | Prof. David Dunn | d.dunn@ucl.ac.uk |
| Statistician | Dr Anna Tostevin | a.tostevin@ucl.ac.uk |
| Co-Investigator | Dr. Alejandro Arenas-Pinto | a.arenas-pinto@ucl.ac.uk |
| Co-Investigator | Dr. Richard Gilson | r.gilson@ucl.ac.uk |
| Co-Investigator | Dr Christopher Rookyard | christopher.g.rookyard@kcl.ac.uk |

**Chief Investigator and PRINCIPAL INVESTIGATOR OF THE SITE**

| Dr Sarah Pett  UCL Centre for Sexual Health and HIV Research  The Mortimer Market Centre  off Capper Street; London, WC1B 6JB | Tel:  Email: | 020 7679 6199 |
| --- | --- | --- |
|  |  | [s.pett@ucl.ac.uk](mailto:a.milinkovic@ucl.ac.uk) |

**SCIENTIFIC ADVISOR**

| Dr Ana Milinkovic | Ana.Milinkovic@chelwest.nhs.uk |
| --- | --- |

**Co-investigators**

| Dr Amelia Moore | Osteoporosis Unit, 1st Floor Tower Wing, Guy’s Hospital, Great Maze Pond, London SE1 9RT |
| --- | --- |
| Dr Christopher Rookyard |  |
| Dr Alejandro Arenas-Pinto; Dr Richard Gilson | UCL Centre for Sexual Health and HIV Research, The Mortimer Market Centre, off Capper Street, London, WC1E 6JB |

**Summary of STUDY**

| **Summary Information Type** | **Summary Details** |
| --- | --- |
| **Short Title of Study** | PETRAM Study |
| **Version** | 4.0 |
| **Date** | 09 May 2021 |
| **EudraCT #** | 2017-000677-36 |
| **REC #** | 17/LO/2018 |
| **Clinical Trials.gov** | NCT03405012 |
| **Study Design** | This is a single centre, 48 week study to explore the pathogenesis of HIV treatment related bone disease by using a novel imaging technique, 18F-Fluoride Positron Emission Tomography/Computed Tomography (18F-PET/CT), which measures regional bone formation. The study will include other standard methods (serum bone markers and DXA) for comparison. Patients enrolled will have baseline, mid-study and end-of-study assessment, with baseline being the date of replacing tenofovir disoproxil fumarate (TDF) in their HIV treatment regimen with tenofavir alafenamide fumarate (TAF), compared to a control group continuing TDF. Allocation to change to TAF or continue TDF will be randomised to allow unbiased assessment of bone changes. |
| **Type of Participants to be Studied** | HIV-1-infected men aged 40-65 years of age, on an ART regimen containing rilpivirine (RPV)/TDF/ emtricitabine (FTC) for at least 6 months, with plasma HIV RNA (pVL) <50cp/mL and without any known history of osteoporosis. |
| **Setting** | Single clinical site: the Mortimer Market Centre, London, UK |
| **Groups to be Compared** | **Group 1 (TAF)**: Change ART to RPV/TAF/FTC (as the fixed dose combination; Odefsey® one tablet daily);  **Group 2 (TDF):** Continue current ART, RPV/TDF/FTC (as the fixed dose combination; Eviplera® one tablet daily). |
| **Study Hypothesis** | We hypothesise that there will be ongoing subclinical loss of bone at the hip and lumbar spine as measured by 18F-PET/CT in those remaining on TDF while in those changing to TAF there will be reversal of some of this subclinical loss on repeat imaging at the end of the study. This study is intended to determine if the improvement in bone mineral density is associated with an increase in bone formation (detected by a change in 18F-PET/CT measurement), or a reduction in bone loss (increase in bone mineral density but no change in 18F-PET/CT). |
| **Primary Outcome Measure(s)** | Change in regional bone formation at the hip and lumbar spine as measured by 18F-PET/CT between baseline and the last study scan. |
| **Secondary Outcome Measure(s)** | Change in regional bone formation at the hip and lumbar spine as measured by 18F-PET/CT at the mid-study scan. |
| **Exploratory Outome Measure(s)** | 1. To compare changes in volumetric bone formation measured by 18F-PET/CT between baseline, mid-point and last study scans. 2. To compare changes in bone mineral density at the hip as measured by DXA vs. 18F-PET/CT between baseline, mid-point, and last study scans. 3. To compare changes in bone mineral density at the lumbar spine as measured by DXA between baseline, mid-point, and last study scans. 4. To compare the changes in bone biomarkers with changes in 18F-PET/CT between baseline, mid-point, and last study scans. 5. To compare the changes in Trabecular Bone Score (TBS) and whole body (lean and fat mass measurements) from DXA between baseline, mid-point and last study scans. 6. To determine the changes in FRAX® score from baseline to the last DXA scan. |
| **Nos. of Participants** | 30 |
| **Duration** | 22-103 weeks (duration variable because of challenges presented by COVID-19 in 2020-2021) |
| **Sponsor** | UCL |
| **Countries of Recruitment** | UK |
| **Funder** | Gilead Sciences |
| **Chief Investigator** | Sarah Pett |

**Abbreviations**

18F-PET/CT 18F-fluoride Positron Emission Tomography

AE Adverse Event

AR Adverse Reaction

AIDS Acquired Immunodeficiency Syndrome

ART Antiretroviral therapy

BMI Body Mass Index

BMD Bone mineral density

cART Combination Antiretroviral Therapy

CI Chief Investigator

COVID-19 Coronavirus Disease 2019

CRF Case Report Form

CT Computed Tomography

CTA Clinical Trial Authorisation

DSUR Development Safety Update Report

DXA Dual-energy X-ray absorptiometry

eGFR Estimated glomerular filtration rate

FBC Full blood count

FRAX Fracture Risk Assessment Tool

FTC Emtricitabine

GCP Good Clinical Practice

HBV Hepatitis B Virus

HCV Hepatitis C Virus

HIV-1 Human Immunodeficiency Virus Type 1

KCL King’s College London

Ki plasma clearance of 18F-fluoride to bone tissue

mSv millisieverts

PI Principal Investigator

PIS Participant Information Sheet

PET Positron Emission Tomography

rpm Revolutions per minute

QA Quality Assurance

QC Quality Control

R&D Research and Development

REC Research Ethics Committee

RPV Rilpivirine

SAE Serious Adverse Event

SAP Statistical Analysis Plan

SARS-CoV-2 Severe Acute Respiratory Syndrome Coronavirus -2

SoC Standard of Care

SPC Summary of Product Characteristics

SUV Standardised Uptake Value

TAF Tenofovir alafenamide fumarate

TBS Trabecular bone score

TDF Tenofovir disoproxil fumarate

TMF Trial Master File

TMG Trial Management Group

TMT Trial Management Team

vBMD Volumetric BMD

VL HIV Viral Load

UCL University College London

UPCR Urine Protein: Creatinine Ratio

**TABLE OF CONTENTS**

1. **BACKGROUND**…………………………………………………………………………………………………………………………………….. 9
   1. 18F-FLUORIDE PET/CT SCANNING PLATFORM………………………………………………………………………………. 9
2. **STUDY OBJECTIVES** ……………………………………………………………………………………………………………………………… 10
3. **STUDY DESIGN**........................................................................................................................................... 11
4. **SELECTION OF PARTICIPANTS**…………………………………………………………………………………………………………….…11

4.1 INCLUSION CRITERIA ……………………………………………………………………………………………………….…………..… 11

4.2 EXCLUSION CRITERIA ……………………………………………………………………………………………………………………… 11

4.3 NUMBER OF PARTICIPANTS……….…………………………………………………………………………………………………... 12

1. **REGULATION AND RECRUITMENT** …………………………………………………………………………………………………….… 12
2. **STUDY ASSESSMENTS AND PROCEDURES** …………………………………………………………………………………………… 13
   1. VISIT SCHEDULE ………………………………………………………………………………………………………………………….… 12
   2. CONTINGENCY MEASURES IMPLEMENTED IN RESPONSE TO THE COVID-19 PANDEMIC……………….. 14
   3. PETRAM TRIAL SCHEME ……………….…………………………………………………………………………………………….… 15

6.4 18F-FLUORIDE PET/CT SCAN ANALYSIS …………………………….………………………………….………………………. 16

6.5 BIOCHEMICAL MARKERS OF BONE TURNOVER ………………………………………….………….……………………... 16

6.6 DXA+TBS ……………………………………………………………………………………………….……………….……..……………… 17

6.7 STUDY MEDICATION AND RISK REDUCTION…………………………………………………….………………………………17

1. **SAFETY REPORTING** …………………………………………………………………………………………………………………………….. 18

7.1 RADIATION EXPOSURE …………………………………………………………………………………………………………………… 18

7.2 SPONSOR RESPONSIBILITIES…………………………………………………….…………………………………………………..… 18

1. **STATISTICAL CONSIDERATIONS** …………………………………………………………………………………………………………… 19

8.1 METHOD OF RANDOMISATION ……………………………………………………………………………………………………… 19

8.2 OUTCOME MEASURES …………………………………………………………………………………………………………………... 19

8.2.1 IMPACT OF COVID-19……………………………………………………………………………………………………………………20

8.3 SAMPLE SIZE …………………………………………………………………………………………………………………………..……… 21

8.4 ANALYSIS ……………………………………………………………………………………………………………………………………….. 21

8.5 INTERIM ANALYSIS ……………………………………………………………………………………………………………………….… 21

1. **REGULATORY AND ETHICAL ISSUES**…………………………………………………………………………………………………… 21

9.1 COMPLIANCE……………………………………………………………………………………………………………………………….…. 21

9.2 DATA TRANSFER……………………………………………………………………………………………………………………………... 22

9.3 ETHICAL CONDUCT OF THE STUDY……………………………………………………………………………………………….…. 22

1. **INDEMNITY** …………………………………………………………………………………………………………………………………………. 22
2. **FINANCE** ……………………………………………………………………………………………………………………………………………… 22
3. **RECORD KEEPING AND ARCHIVING** …………………………………………………………………………………………………….. 22
4. **OVERSIGHT AND STUDY COMMITTEES** ………………………………………………………………………………………………. 23
5. **PUBLICATION POLICY** ……………………………………………………………………………………………………………………….…. 23
   1. INTELLECTUAL PROPERTY………………………………………………………………………………………………………………23
6. **REFERENCE LIST**………………………………………………………………………………………………………………………………..… 25

**1.** **BACKGROUND**

# The life expectancy of HIV-infected patients continues to improve due to antiretroviral therapy (ART). However, one of the side-effects of ART, particularly regimens containing the drug tenofovir disoproxil fumarate (TDF), is bone loss [1]. This bone loss is of concern because patients are starting treatment earlier, the HIV-positive cohort is ageing, and patients will be exposed to ART over very long periods.

# Our understanding of the pathogenesis of bone loss in HIV-infected patients on ART is incomplete. Moreover, there are likely to be several contributing factors including age-related bone loss, vitamin D deficiency, corticosteroid-induced loss, nutritional deficiency, as well as specific ART effects. Teasing out these different and/or multiple pathways leading to bone loss is critically important, as it will allow us to refine the diagnosis of the type of bone disease in a patient, ensuring that the correct treatment is given, and in some cases avoiding unnecessary changes in ART. Currently available methods to assess bone disease are limited to serum bone markers which provide a global assessment of bone turnover, bone biopsies which are limited to a single biopsy site and too invasive for widespread or repeated assessments, and changes in bone mineral density (BMD) measured using dual-energy X-ray absorptiometry (DXA). DXA gives 2-dimensional information, which correlates with fracture risk at the hip and spine, but it cannot elucidate the exact mechanism of the bone loss at those sites. In particular, it is unable to measure bone turnover at trabecular and cortical rich sites.

# 18F-fluoride positron emission tomography/computed tomography (18F-PET/CT), a novel functional imaging technique, allows the non-invasive quantitative assessment of bone mineralisation at specific clinically-important fracture sites [2]. It provides much more information than simple measures of bone mineral density, such as DXA, and offers several advantages over bone biopsy, and bone turnover markers, because it allows a direct measurement of bone formation at sites such as the spine and hip [3]. This allows an insight into the changes in bone turnover which may lead to an increased risk of fracture at these sites.

#

# **1.1. 18F-FLUORIDE PET/CT SCANNING PLATFORM**

#

# 18F-PET/CT imaging allows the quantitative assessment of regional bone formation – SUV and Ki. SUV is a measure of bone metabolic activity that assumes uniform distribution of the scan tracer whilst ki is a measure of bone metabolic activity and mineralisation that incorporates the blood concentration of the trace. Ki is measured in units of mL.min-1.mL-1 [4, 5]. The technique has been validated in the non-HIV setting by comparison with bone histomorphometric indices with significant correlations observed between regional skeletal kinetic parameters using 18F-PET/CT and the bone formation and mineral apposition rate [6, 7]. Furthermore, 18F- PET/CT has been developed and validated by Dr Amelia Moore’s group at KCL as a tool to investigate regional bone metabolism in subjects with metabolic bone disease [8-10] and evaluate the response to current and novel therapies [11-13]. The method has shown important differences in remodelling activity between cortical and trabecular rich sites [14]. The technique is highly acceptable to patients, with short scan times and radiation exposure well within acceptable limits as set by the UK guidelines from the Administration of Radioactive Substances Advisory Committee (ARSAC) [15].

# TDF is a prodrug of the nucleotide analogue tenofovir (TFV). Tenofovir alafenamide (TAF) is a phosphonoamidate prodrug of TFV, which undergoes conversion to TFV intracellularly, achieving higher active metabolite concentrations in peripheral blood mononuclear cells and lower plasma TFV exposures than TDF. TAF has demonstrated significantly less impact on renal and bone markers, compared to TDF [16-18]. Study GS-292-0109 showed that switching away from a TDF-containing regimen to a TAF-containing regimen resulted in significant improvements in spine and hip BMD as measured by DXA. In those switching to TAF vs. those remaining on TDF, BMD increased at both the spine (+1.79% vs. -0.28%; p<0.001), and the hip (+1.37% vs. -0.26%; p<0.001), respectively [19].

# Our study is novel as we believe this is the first time that 18F-PET/CT will be used in HIV-infected patients. Importantly, this novel imaging technique has the capacity to provide important insights into the pathogenesis of HIV-treatment-related bone disease and longitudinal changes over time can be quantified. In HIV-infected individuals different types of bone disease may coexist and different aetiologies may be more readily differentiated than by conventional methods such as DXA. By using 18F-PET/CT it is hoped that it will be possible to determine if the improvement in bone density expected to be seen with changing patients to TAF, as compared to continuing with TDF, is associated with an increase in bone formation, detected by a change in 18F-PET/CT measurement, or a reduction in bone loss. In the latter case, there would be an increase in BMD but no change in 18F-PET/CT.

# The rationale for restricting this study to males is to avoid heterogeneity. Women are more likely than their male counterparts to have osteopenia/osteoporosis and may be taking treatment (e.g. HRT or bisphosphonates) which can affect bone metabolism.

**2. STUDY OBJECTIVES**

**2.1. PRIMARY OBJECTIVE**

- To determine the change in regional bone formation at the hip and lumbar spine, as measured by 18F-PET/CT between baseline and last study scan* in patients starting a TAF-based ART regimen compared with those continuing a TDF-based regimen.

**2.2. SECONDARY OBJECTIVE**

- To determine the change in regional bone formation at the hip and lumbar spine as measured by 18F-PET/CT between baseline and mid-point scan** in patients starting a TAF-based ART regimen compared with those continuing a TDF-based regimen;

**2.3 EXPLORATORY OBJECTIVE**

- To compare changes in the volumetric bone formation at the hip and lumbar spine as measured by 18F-PET/CT between baseline, mid-point and last study scans.
- To compare changes in bone mineral density at the hip as measured by DXA vs. 18F-PET/CT between baseline, mid-point, and last study scans.
- To compare changes in bone mineral density at the lumbar spine as measured by DXA between baseline, mid-point, and last study scans.
- To compare the changes in bone biomarkers with changes in 18F-PET/CT between baseline, mid-point, and last study scans.
- To compare the changes in Trabecular Bone Score (TBS) and whole body (lean and fat mass measurements) from DXA between baseline, mid-point and last study scans;
- To determine the changes in FRAX® score from baseline to the last DXA scan.

*last study scan= the final scan on study, and ‘approximates’ to the week 48 visit; this timepoint was variable because of the challenges presented by COVID-19, the final scan was anywhere between 22-103 weeks.

**mid-point scan= the scan midway between baseline at t=0, and the final scan on study; and ‘approximates’ to the week 24 visit.

# **3. STUDY DESIGN**

This study is designed to explore the pathogenesis of HIV treatment related bone disease by using a novel imaging technique (18F-PET/CT) which measures regional bone formation. The study will include other standard methods (serum bone markers and DXA) for comparison. Patients enrolled will have baseline, midpoint (‘approximates’ to the week 24 visit) and final (‘approximates’ to the week 48 visit) scan assessment. Baseline will be the date of the baseline 18F-PET/CT as this also correlates to the date of replacing a TDF based regimen with a TAF-based regimen, compared to a control group continuing TDF. Treatment allocation will be randomised to allow an unbiased assessment of bone changes.

# **4. SELECTION OF PARTICIPANTS**

There will be no exceptions to eligibility requirements at the time of recruitment. Questions about eligibility criteria should be addressed prior to attempting to enrol the participant.

The eligibility criteria for this study have been carefully considered. The eligibility criteria are the standards used to ensure that only medically appropriate patients are considered. Patients not meeting the criteria should not join the study. For the safety of the patients, as well as to ensure that the results can be useful for making treatment decisions regarding other patients with similar diseases, it is important that no exceptions be made to these criteria for admission to the study.

Participants will be considered eligible for enrolment in this study if they fulfil all the inclusion criteria and none of the exclusion criteria.

## **4.1. INCLUSION CRITERIA**

## 1. HIV-1-infected men aged from ≥40 to 65 years on ART regimen containing RPV/TDF/FTC for ≥6 months;

## 2. Virologically suppressed (<50 cp/mL) for >24 weeks;

## 3. No known history of osteoporosis (defined as a T-score > -2.5 at the lumbar spine, femoral neck or total hip using DXA);

## 4. Willing to change ART regimen as above for the duration of the study;

## 5. No reason, in the opinion of the investigator, except for the purpose of the study, to immediately replace TDF with TAF in their regimen;

## 6. Willing to comply with study procedures.

## **4.2 EXCLUSION CRITERIA**

## 1. Contraindication to the receipt of TAF;

## 2. Contraindication to 18F-PET/CT scanning e.g. not able to lie still for 25 minutes on the scanner or any metal inserts in the hip or spine regions;

## 3. Anticipated to require additional radiological imaging during the 48 weeks of study participation which will mean the total cumulative ionising radiation dose is >50 millisieverts (mSv) over the course of the study;

## 4. Current or previous treatment (within the prior 12 months) which can affect bone metabolism including exposure to anabolic steroids, oral glucocorticoids (for >3 months at a dose of prednisolone of ≥5mg daily), and bisphosphonates;

## 5. Active hepatitis C coinfection (determined by HCV Ag or RNA test within the last 24 weeks).

## **4.3. NUMBER OF PARTICIPANTS**

A total of 30 participants will be recruited at a single clinical site, Mortimer Market Centre.

# **5. REGULATION AND RECRUITMENT**

Written informed consent to enter into the study must be obtained from participants after explanation of the aims, methods, benefits and potential risks of the study and **BEFORE** any study-specific procedures are performed (see Patient information Sheet (PIS) and Consent form).

Prior to the screening visit, the study will be discussed with any potentially suitable patients at their routine clinic visits and the PIS given to them so that they have at least 24 hours to consider participating in the study.

**5.1 SCREENING PROCEDURES & PRE-RANDOMISATION INVESTIGATIONS**

The screening visit and randomisation visit can be conducted on the same day, providing that results of blood tests done as standard-of-care are available to confirm eligibility. If the results of blood tests required to confirm eligibility are not available, a separate screening visit must be conducted.

Written informed consent to enter into the trial must be obtained from patients after explanation of the aims, methods, benefits and potential hazards of the trial and BEFORE any trial-specific procedures are performed or any blood is taken for the trial (see Consent Form).

It must be made completely and unambiguously clear that the patient is free to refuse to participate in all or any aspect of the trial, at any time and for any reason, without incurring any penalty or affecting their treatment.

Signed consent forms must be kept by the investigator and documented in the case record forms (CRF) held at site and a copy given to the patient. With consent, a letter should be sent to the general practitioner informing him/her of the trial and the patient's involvement in it.

Signed consent forms must be kept by the investigator and documented in the CRF folder and a copy given to the participant as per ICH-GCP requirements.

**5.2 RANDOMISATION**

Eligibility will be confirmed via the Screening CRF and patients randomised to:

- **Group 1 (TAF)**: Change ART to RPV/TAF/FTC (as the fixed dose combination; Odefsey® one tablet daily);
- **Group 2 (TDF):** Continue current ART, RPV/TDF/FTC (as the fixed dose combination; Eviplera® one tablet daily).

Randomisation will be computer-generated. Randomisation will be conducted through the Research Department of Infection and Population Health UCL Institute for Global Health.

Randomisation may occur on any day of the week.

# **6. STUDY ASSESSMENTS AND PROCEDURES**

Patients enrolled in this study will need to attend four additional appointments over and above their regular clinic visits. These additional appointments include attendance at the PET Centre and the Osteoporosis Unit at Guy’s and St. Thomas’s Hospital, where all the additional scans (18F- PET/CT and DXA) will be performed at baseline, mid-study (‘approximates’ to the week 24 visit) and at the end of the study (‘approximates’ to the week 48 visit).

**18F-fluoride PET scan:** A 25-minute static 18F- PET/CT scan of the lumbar spine and hip at baseline (**Visit 1b**), mid-study (‘approximates’ to the week 24 visit, **Visit 2b**) and at the end of the study (‘approximates’ to the week 48 visit, **Visit 3b**). The procedure will involve an injection of 90 MBq 18Fluoride, followed by a 25-minute static 18F- PET/CT scan (4-bed position) from the lumbar spine to mid-femur 1 hour after the injection. Three venous blood samples will be collected from an indwelling cannula at 30 minutes post-injection immediately prior to and immediately after the static scan to measure 18F activity in plasma; the arterial input function will be derived using a semi-population input function method. The images will be acquired using a PET/CT system with a 17-cm axial field of view and a CT scan will be acquired prior to the PET scan for attenuation correction of the PET images, and to define the bone anatomy for the placement of the regions of interest for the PET scan analysis. A low dose CT scan will be applied for attenuation correction at the four bed positions and a higher dose CT scan will be required for the volumetric BMD (vBMD) measurements at the hip. vBMD will be measured at the hip by scanning a hydroxyapatite reference phantom so that the Ki values in this region can be corrected for vBMD. The Ki values at the lumbar spine will be corrected for areal BMD from the DXA scan at the lumbar spine. The PET measurements will be corrected for the partial volume effect using recovery coefficient data acquired previously following a rod phantom experiment and theoretical modelling [8]. The PET/CT scanner will undergo a recalibration procedure approximately every three months as a quality control process that is standard practice for maintenance of the scanner.

**DXA scan:** All subjects will have a DXA scan of BMD at the lumbar spine, non-dominant hip and whole body at baseline (**Visit 1b**), mid study (‘approximates’ to the week 24 visit, **Visit 2b**) and at the end of the study (‘approximates’ to the week 48 visit, **Visit 3b**) to assess change in BMD in response to treatment. For individuals who have a baseline DXA scan showing osteoporosis of either the hip or lumbar spine, they will be withdrawn from the study and return to follow-up by their usual HIV physician. The site team will recommend a review of whether a TAF-based regimen or switch from TDF is appropriate, and to consider bisphosphonate treatment/assessment of bone health lifestyle factors.

**Blood samples** (for routine pathologies and stored samples) will be collected at baseline (**Visit 1a**), weeks 24 (**Visit 2a**) and 48 (**Visit 3a**). Samples for future bone biomarker analyses will be stored at Mortimer Market Centre.

- 1. **VISIT SCHEDULE**

| **Visit 1a – Screening/ Baseline (Day 0) visit at** **Mortimer Market Centre:**  1) Informed consent obtained;  2) Eligibility check;  3) Completion of screening procedures including:   - Participant will complete a 7-day recall adherence questionnaire; - Record height and weight and vital signs; - Complete medical and HIV history; - Review AE and concomitant medications; - Calculate FRAX score; - Fasted blood and urine samples to assess, plasma HIV RNA and T-cell subsets, full blood count, renal function, liver function tests, bone panel (calcium, phosphate), uPCR, vitamin D, storage sample for future assessment of bone biomarkers; - Provide details of the baseline week 0 (**Visit 1b**) scanning visit. Participants will be instructed NOT to change medication until they have attended **Visit 1b**, and also not to reveal at any point to the scanning unit what ART they are taking;   4) Randomisation;  5) Dispense new ART if changing (or repeat prescription as required);  **Visit 1b : Osteoporosis Unit, Guy’s Hospital and PET Centre, St Thomas’ Hospital**  The **Visit 1b** will take place within 14 days of **Visit 1a.**  1) DXA (bone density) scan of the spine, non-dominant hip and whole body (**Section 6.5**);  2) Static 18F-PET/CT bone scan of the hip and lumbar spine (**Section 6.3**). This will involve insertion of a peripheral venous cannula which will remain in situ for the entire PET visit to obtain the three blood draws during the scanning procedure as described in **Section 6.3**.  MMC study staff will contact the participant within 3 working days of this visit to verify the scans occurred as planned, gather AE, and verify the date participants change ART (if applicable).  **Visit 2a – 24 weeks - Mortimer Market Centre, London**  This visit will take place 24 weeks (+14 days) from **Visit 1b**. Procedures include:  1) Participant will complete a 7-day recall adherence questionnaire;  2) Fasted blood samples to assess, plasma HIV RNA, renal function, liver function tests, bone panel (calcium, phosphate), vitamin D, PTH and storage sample for future assessment of bone biomarkers;3) Record adverse events and concomitant medications;  4) Check ART and dispense a further supply of ART as required;  5) Provided details of the week 24 (**Visit 2b**) scanning visit;    **Visit 2b – Mid-scan (‘approximates’ to the 24 week visit) - Osteoporosis Unit, Guy’s Hospital and PET Centre, St Thomas’ Hospital**  This visit will take place within 42 days (6 weeks) of **Visit 2a**. Procedures will be identical to those conducted at **Visit 1b**. MMC study staff will contact the participant within 3 days of this visit to verify the scans went ahead as planned; gather AE.  **Visit 3a – 48 weeks - Mortimer Market Centre, London**  This visit will take 24 weeks (+ 14 days) after **Visit 2b**.  1) Participant will complete a 7-day recall adherence questionnaire;  2) Fasted blood and urine samples to assess, plasma HIV RNA, T-cells, FBC, renal function, liver function tests, bone panel (calcium, phosphate), uPCR, vitamin D, storage sample for future assessment of bone biomarkers;  3) Calculate FRAX score;  4) Record adverse events and concomitant medications;  5) Check ART and dispense a further supply of ART – sufficient to continue at least until after visit **3b** is scheduled. ART after Visit **3b** is at the discretion of the investigator (return to standard of care, which may include continuation of TDF- or TAF-based ART);  6) The next routine visit in the clinic will be booked with the participant’s usual clinician;  7) Provided details of the week 48 (**Visit 3b**) scanning visit.  **Visit 3b – last scan (‘approximates’ to the 48 week visit, Final study visit) - Osteoporosis Unit, Guy’s Hospital and PET Centre, St Thomas’ Hospital**  This visit will take place within 42 days of **Visit 3a**. The visit is identical to the prior scanning visits (**Visits 1b and 2b**). MMC study staff will contact the participant within 3 days of this visit to verify the scanning visit went to plan and gather AE and ensure all arrangements are in place for a routine clinic follow-up appointment. Participants will be informed of the timelines to receive the final results of the study.  **6.2. CONTINGENCY MEASURES IMPLEMENTED IN RESPONSE TO THE COVID-19 PANDEMIC**  In response to the evolving COVID-19 pandemic, on 18^th^ March 2020 suspension of all outstanding trial face-to-face follow-up visits, DEXA scans, and PET/CT scans was implemented. This was to comply with the national public health guidance on preventing onward transmission of SARS-CoV-2 by minimising non-essential exposures for participants and trial staff. No safety concerns associated with the study drugs were identified given both are being used within their licensed indication.  Recruitment had completed prior to the onset of the pandemic. Participants still in follow-up will each be contacted to verify ongoing consent in the case of the study being extended by up to six months as a result of the suspension. Scheduled visits continued over the phone to assess for adverse events, concomitant medication changes, and supply of ART. Study drug will continue to be supplied throughout the suspension period to prevent treatment interruptions, utilising existing home delivery services or couriers as required. If any participants contract COVID-19 warranting hospital admission, in addition to usual SAE reporting, investigators will review any additional imaging that occurs to ensure the total radiation dosage does not exceed the exclusion threshold.  Two consequences of the COVID-19 strategy are that some participants will take the study drugs for an extended duration and there will be discordance between the ongoing scheduled telephone study visits and their respective scans which will only occur when radiology activity resumes. As a result, the collection of outstanding fasted blood and urine samples, as stated in the Visit Schedule, will take place prior to any delayed scans as an unscheduled visit. The primary analysis is intention-to-treat and so will be unaffected, but sensitivity analyses will account for the extended duration of treatment for some participants. Further details will be provided in the Statistical Analysis Plan. |
| --- |

**6.3. PETRAM TRIAL SCHEME**

| **Study week number** | **Visit 1aφ**  **SCRN/Baseline (MMC)** | **Visit 1b**  **(week 0)***  **(Guys/STH)** | **Visit 2a**  **(week 24)**  **(MMC)** | **Visit 2b**  **(week 24)***  **(Guys/STH)** | **Visit 3a**  **(week 48)**  **(MMC)** | **Visit 3b**  **(week 48)***  **(Guys/STH)** |
| --- | --- | --- | --- | --- | --- | --- |
| **Clinical Assessments** |  |  |  |  |  |  |
| Informed consent ^a^ | • |  |  |  |  |  |
| Review eligibility | • |  |  |  |  |  |
| Complete medical/HIV history | • |  |  |  |  |  |
| AEs, concomitant medication | • |  | • |  | • |  |
| 7-day adherence recall | • |  | • |  | • |  |
| FRAX score | • |  | • |  | • |  |
| Height/weight | • |  |  |  |  |  |
| Dispense ART | • |  | • |  | • |  |
| **Lab and radiology Assessments** | **Visit 1a** | **Visit 1b** | **Visit 2a** | **Visit 2b** | **Visit 3a** | **Visit 3b** |
| Fasted biochemistry ^b^ | • |  | • |  | • |  |
| Renal function ^c^ | • |  | • |  | • |  |
| Urine PCR | • |  |  |  | • |  |
| Haematology (FBC and diff) | • |  |  |  | • |  |
| T cells (CD4+ and CD8+ absolute& %) | • |  |  |  | • |  |
| Virology: plasma HIV-1 RNA | • |  | • |  | • |  |
| HCV Ag | • |  | • |  | • |  |
| DXA scan of lumbar spine, non-dominant hip, whole body and TBS |  | • |  | • |  | • |
| Static 18F-PET/CT bone scan (hip and spine) |  | • |  | • |  | • |
| **Storage Samples** | **Visit 1a** | **Visit 1b** | **Visit 2a** | **Visit 2b** | **Visit 3a** | **Visit 3b** |
| Fasted blood samples for future biomedical markers of bone turnover ^d^ | • |  | • |  | • |  |

1. Participants will have received the PIS at least 24 hrs before this screening/baseline visit to ensure they have had enough time to read this in full
2. Fasted (≥8hrs) biochemistry: bone screen to include calcium, phosphate; liver function tests: to include ALT, ALP, albumin, total bilirubin; parathyroid hormone (PTH), vitamin D;
3. Renal function: creatinine and urea;
4. Fasted Biomedical markers of bone turnover.

**φ** Screening and randomisation can be done at the same visit, provided historic blood samples done as part of the participant’s standard-of-care are available to confirm eligibility. If these are not available, then a separate screening and randomisation (Day 0) visit will be conducted.

* see above for the visit windows for the scan visits.

## **6.4. 18F-FLUORIDE PET/CT SCAN ANALYSIS**

Regions of interest will be applied to including but not limited to the femoral neck, intertrochanteric region, total hip, each lumbar vertebral body, and upper femoral shaft for the calculation of SUV and Ki which reflect regional bone perfusion and bone turnover.

The 18F-PET scans will be analysed on an ongoing basis by the Imaging Scientist Dr Christopher Rookyard and checked by Professor Glen Blake, both of whom have significant experience in this scan analysis. As a quality control measure, the first scan will be assessed for image quality prior to the study continuing. The PET data will be corrected for attenuation using the CT data and the images reconstructed by filtered back-projection. The hip data will be corrected for vBMD and the spine data corrected for areal BMD. Ki will be determined using the static scan Ki method [20]. For instances where SUV and ki results are highly discordant, PET/CT images will be independently reviewed by two qualified radiologists to assess tracer distribution for that scan.

The ROIs will include but not be limited to:

(1) a cylindrical section of cortical bone in the upper femoral shaft measured from just below the lesser trochanter (Femoral shaft ROI).

(2) a region of mixed trabecular and cortical bone between the femoral neck and lesser trochanter anatomically equivalent to the total hip ROI used in DXA scanning (Total hip ROI);

(3) a region between the lesser and great trochanter (Intertrochanteric ROI);

(4) a region of trabecular bone in the femoral neck with its orientation similar to that defined in DXA scanning (Femoral neck ROI);

(5) for the static scan analysis of the lumbar spine, an elliptical ROI will be placed in the middle of each vertebra L1 to L4 excluding the end plates on the corresponding CT segmented scan. Each PET frame will be aligned to the corresponding CT scans and the final lumbar spine ROI will be based on the average of the four individual vertebrae (Lumbar spine ROI).

Radiation dose for each visit:

1) Dose from 90MBq 18F-fluoride injection: 1.55 mSv

2) Dose from low dose CT (20mA) for 4 bed positions to include lumbar spine (scan length of 51cm) (including the 20% increase which is conducted on the MPE reports): 0.83 mSv

3) Dose from high dose CT (100mA) for Hip and Femur (including the 20% increase): 1.8 mSv

Total per visit: 4.18 mSv

## **6.5. BIOCHEMICAL MARKERS OF BONE TURNOVER ON STORED SAMPLES**

Approximately 16 mL of blood will be collected in 1 x 5 mL SST tube, 1 x 6mL EDTA and 1 x 5mL Lithium Heparin tube from subjects in a fasting state. The samples will be allowed to stand for approximately 10 minutes at room temperature, and then centrifuged for 10 minutes at approximately 2500 rcf. Immediately after centrifugation serum/ plasma from each tube will be placed into 2 vials labelled with the subject’s unique study ID, visit number, date and frozen at -70°C.

The bone markers to be analysed will be procollagen Type I N terminal propeptide (PINP) and cross-linked C telopeptides of Type I collagen (CTX). This analysis will be performed at a specialist research lab with the appropriate facilities and experience to produce validated results. Any remaining sample volume will be retained and can be considered for further biomarker analysis in the future.

**6.6. DXA+TBS**

Dual-energy x-ray absorptiometry (DXA) scans will be performed at baseline (**Visit 1b**), mid-study (‘approximates’ to the week 24 visit, **Visit 2b**), and at the end of the study (‘approximates to the week 48 visit, **Visit 3b**) to evaluate the change in areal BMD (in g/cm2) in response to treatment at the lumbar spine (L1-L4), total hip, femoral neck, trochanter, intertrochanteric, and whole body using standard protocols. Daily instrumental quality control of the DXA scanner will be performed using the manufacturer’s anthropomorphic spine phantom. The follow-up scans will be performed on the same scanner that the baseline scan was performed.

The Fracture Risk Assessment Tool (FRAX) using the subject’s clinical risk factors and femoral neck BMD will be applied to assess the change in FRAX between baseline and the last DXA scan.

Loss of subcutaneous abdominal tissue (SAT) with sparing of visceral adipose tissue (VAT) has been documented in HIV + men on ART, therefore, lean and fat mass measurements will be calculated from the whole body DXA scans and assessed between baseline, mid-scan, and the final scan (using software Apex 4 upwards).

Trabecular bone score (TBS) is a novel grey-level textural analysis applied to the lumbar spine DXA images, to indirectly estimate trabecular microarchitecture. TBS will be measured between baseline, mid-scan, and final scan.

Scan analysis: Scans should be performed on array mode on GN63 scanner.

DXA radiation dose for each visit:

DXA scan spine and hip 0.01 mSv

DXA scan whole body 0.0042 mSv

Total per visit: 0.0142 mSv

**6.7.** **STUDY MEDICATION AND RISK REDUCTION**

Medication will be supplied according to standard procedures from local pharmacy. This includes all drugs for the control arm and Odefsey® for the experimental group which will be provided from pharmacies stock. Odefsey® will be supplied by Gilead Sciences and delivered to Mortimer Market Centre pharmacy department and will be managed by local pharmacy.

As a measurement of risk minimisation following procedures will be in place:

1. Odefsey® should be stored securely in a dedicated pharmacy unit.
2. Odefsey® should be clearly segregated from other stock to facilitate accountability and labelled as patient and trial specific.
3. Accountability records are to be maintained by delegated staff to accurately to capture the receipt and dispensing of Odefsey® (strength, batch number, expiry, as well as reflect the movement of both products from supplier to participant administration to allow traceability and recall if required.

**7. SAFETY REPORTING**

The principles of ICH GCP require that both investigators and Sponsors follow specific procedures when notifying and reporting adverse events or reactions in clinical studies. Therefore, any drug-related adverse event will be reported and followed according to the PETRAM procedures. All SAEs will be reported to Gilead Sciences to the extent required by the contractural arrangements between UCL and Gilead Sciences. An Independent Clinical Reviewer at UCL, Dr John Saunders, will review all SAE documentation for completeness prior to submission.

**7.1. RADIATION EXPOSURE**

PET is considered to be a safe examination with no known side effects from the 18-Fluoride tracer. However, the DXA and 18F-PET scans involve exposure to radiation. The total effective dose associated with the 18F-PET/CT and DXA scans is 12.6 mSv, which is equivalent to the dose a person in the UK receives from natural background radiation (2.2 mSv/a (millisievert per year) over approximately 5.7 years. The radiation dose associated with the DXA scans is negligible and equivalent to just a few days background radiation. Radiation dose associated with the 18F-PET/CT scans has been kept to a minimum by using a dose of 18F-fluoride (90 MBq) that is one-third of the dose typically used in clinical practice. Furthermore, the dose associated with the CT scan for the four bed position has been kept to a minimum by using a low tube current for attenuation correction and image segmentation. For comparison, the total effective radiation dose each participant will receive in this study is slightly less to that received by having two CT Chest examinations.

Adverse events will be recorded at all study visits and subjects will be withdrawn from the study if they or the investigator have safety or tolerability concerns, as perceived by the investigator or subject. Any serious adverse events (SAE), Serious Adverse Reaction (SAR) or Unexpected Serious Adverse Reaction (USAR) will be recorded at each study visit and reported as appropriate.

SAE REPORTING

Following the standard PETRAM procedures.

Within 24 hours of becoming aware of an SAE, please email
the completed SAE form to the PI of the study [sarah.pett@nhs.net](mailto:sarah.pett@nhs.net)

## **7.2. SPONSOR RESPONSIBILITIES**

Medically-qualified staff at the coordinating centre and/or the Chief Investigator (or a medically-qualified delegate) as well as independent clinical reviewer at UCL will review all SAE reports received. The causality assessment given by the local investigator at the hospital cannot be overruled; in the case of disagreement, both opinions will be provided in any subsequent reports.

The Sponsor will delegate responsibility for the overall management of the study to the coordinating centre and the PETRAM project management. The PETRAM project management team is responsible for the reporting of SAEs to the Research Ethics Committee and also keeping all investigators informed of any safety issues that arise during the course of the study.

The PETRAM project management team will submit Annual Safety Reports on the study to the Research Ethics Committee.

# **8. STATISTICAL CONSIDERATIONS**

**8.1. METHOD OF RANDOMISATION**

Patients will be randomised on an individual basis without stratification (because of the small sample size). A computer algorithm will be written by the Trial Statistician to produce a sequentially numbered randomisation list that will achieve close such that perfect balance is achieved between the two treatment arms at the end of the trial (i.e. 15 patients per arm) with a maximum imbalance of 3 patients at any point on the list. This list will be encrypted and accessed by a program that will reveal the next randomisation, but not the whole list, for patients who are entered into the trial. Randomisation will be performed by the Chief Investigator or a delegated individual in case of her non-availability.

**8.2. OUTCOME MEASURES**

**8.2.1. IMPACT OF COVID-19**

As a result of the COVID-19 epidemic, many of the scheduled radiology scans had to be cancelled or postponed, with the scans therefore not taking place exactly within the week 24 and week 48 windows as planned. This has major consequences for the statistical analysis of the study. In particular, the wide variation in the timing of scans means that it is no longer feasible to conduct cross-sectional analyses. Instead, the primary analysis will consider the **last scan performed in the study** for each participant. This ranges between 22 and 103 weeks (Figure 1); the analysis will be adjusted to correct for this variability. It is noted that all participants (other than those who withdrew) remained on their randomised treatment at the time of the latest scan without interruptions.

Two individuals were unable to have their baseline scan within the week 0 window, one randomised to Eviplera and one to Odefsey. As a result, no treatment switch took place for the Odefsey-randomised participant and they continued Eviplera up until the time of their delayed PET scan (occurred approximately around week 24 for both). The delayed PET scan date served as time=0 for these two individuals and they both finished the study after 24 weeks due to the delayed baseline scan.

*Figure 1: histogram showing the occurrence of the last scan for each participant relative to baseline*

**Primary**

Change in regional bone formation at the hip and lumbar spine (SUV, ki) as measured by 18F-PET/CT from baseline until the latest scan timepoint.

**Secondary**

Among participants with two post-baseline scans, change in regional bone formation at the hip and lumbar spine (SUV, ki) as measured by 18F-PET/CT at the mid-point scan.

**Exploratory**

1. Changes in ki/vBMD as measured by 18F-PET/CT for the hip
2. Changes in ki/vBMD as measured by 18F-PET/CT for the lumbar spine with vBMD derived from respective DXA scans
3. Changes in bone mineral density at the hip measured by DXA vs. 18F-PET/CT
4. Changes in bone mineral density at the lumbar spine measured by DXA
5. Changes in bone biomarkers vs. 18F-PET/CT;
6. Changes in Trabecular Bone Score (TBS) measured by DXA.
7. Changes in Whole body fat (lean and fat mass measurements) measued by DXA
8. Changes in FRAX score

**8.3. SAMPLE SIZE**

A total of 30 subjects (in order to have 30 evaluable subjects more than 30 may be randomised) will be randomised in a 1:1 ratio (15 subjects per group) into the two study groups. Assuming a mean increase from baseline of 25% in the plasma clearance of 18F-fluoride to bone tissue (Ki and SUV, primary outcome) in the TAF group and no change in the TDF group, and a standard deviation of 20% [17] then a total of 15 subjects in each group provide at least 90% power to detect a significance difference between groups with a Type-1 error of p=0.05. The actual power may be higher than this since the method of analysis (analysis of covariance, see below) is a more powerful technique than change from baseline.

**8.4. ANALYSIS**

The primary analysis will compare the two groups as allocated (intention to treat, ITT). Analysis of covariance will be used to adjust for baseline value and the time interval between the baseline scan and the final scan. The need to include an interaction term between this time interval and treatment arm will be considered. Ki and SUV values may be transformed to conform with the assumptions in the statistical model (the transformation will be chosen before conducting any analyses stratified by randomised group). Point estimates will be accompanied by 95% confidence intervals. P values will not be adjusted for multiple comparisons [21].

Additional, exploratory analyses will compare the concordance between changes in bone loss as assessed by 18F-PET/CT, DXA, and biochemical markers. Although the different screens were planned to be conducted in the same week, this was also disrupted by the covid-19 epidemic. These analyses will therefore consider comparable screens in a window of ±12 weeks.

**8.5. INTERIM ANALYSES**

No comparative interim analyses will be conducted.

**9. REGULATORY AND ETHICAL ISSUES**

## **9.1. COMPLIANCE**

The study will be conducted in compliance with the approved protocol, the Declaration of Helsinki (fourth revision 1996) the principles of Good Clinical Practice (GCP), the UK Data Protection Act (DPA number Z5886415), the Commission Directive 2005/28/EC with the implementation in national legislation in the UK by Statutory Instrument 2004/1031 and subsequent amendments, and NHS research governance and other regulatory requirements, as appropriate in the participating centres. Each study site will comply with the above.

The site will inform the coordinating centre as soon as they are aware of a possible serious breach of compliance, where a “serious breach” is one that is likely to affect to a significant degree:

- The safety or physical or mental integrity of the subjects in the study, or
- The scientific value of the study

**9.2. DATA TRANSFER (handling, processing and storage)**

CRFs, clinical notes and administrative documentation should be kept in a secure location (for example, locked filing cabinets in a room with restricted access) and held for 20 years after the end of the trial. During this period, all data should be accessible to the competent or equivalent authorities, the Sponsor, and other delegated authorities with suitable notice. The data may be subject to an audit by the competent authorities.

The Chief Investigator will act as custodian for the trial data. The following guidelines will be strictly adhered to:

- Subject data will be anonymised
- All anonymised data will be stored on a password protected computer.
- All trial data will be stored and archived in line with the Medicines for Human Use (Clinical Trials) Amended Regulations 2006 as defined in the Joint Clinical Trials Office Archiving SOP (standard operating procedure).

## **9.3. ETHICAL CONDUCT OF THE STUDY**

Before initiation of the study, the protocol, all informed consent forms, and information materials to be given to the prospective participant will be submitted to an ethics committee for review. Any further amendments will be submitted for review by the ethics committee.

The rights of the participant to refuse to participate in the study without giving a reason must be respected. The participant must remain free to change their mind at any time about their participation in the study without giving a reason and without prejudicing their further treatment and medical care.

# **10. INDEMNITY**

University College London holds insurance against claims from participants for harm caused by their participation in this clinical study. Participants may be able to claim compensation if they can prove that UCL has been negligent. However, if this clinical study is being carried out in a hospital, the hospital continues to have a duty of care to the participant of the clinical study. University College London does not accept liability for any breach in the hospital’s duty of care, or any negligence on the part of hospital employees. This applies whether the hospital is an NHS Trust or otherwise.

# **11. FINANCE**

This study is funded by Gilead Sciences and will be coordinated by the UCL Centre for Sexual Health and HIV Research. A written agreement with the site Principal Investigator and/or the Investigator’s institution and the study sponsor will outline the funding arrangements.

**12.** **RECORD KEEPING AND ARCHIVING**

At the end of the trial, all essential documentation will be archived securely by the CI for a minimum of 20 years from the declaration of end of trial.

Essential documents are those which enable both the conduct of the trial and the quality of the data produced to be evaluated and show whether the site complied with all applicable regulatory requirements.

The sponsor will notify sites when trial documentation can be archived. All archived documents must continue to be available for inspection by appropriate authorities upon request

# **13. OVERSIGHT AND STUDY COMMITTEES**

##

## **13.1 TRIAL MANAGEMENT GROUP**

A trial management group (TMG) that will include the chief investigator, the study statistician and co-investigators, will be formed to conduct the day-to-day management of the study.

# **14. PUBLICATION POLICY**

The results of this PETRAM study will be published following the completion of the study. The individual patient results will be made available to patients through their treating clinicians.

In order to avoid disputes regarding authorship, it is important to establish a consensus approach that will provide a framework for all publications derived in full or in part from this clinical trial. The following approach is derived from the Lancet and from the publication policies used in OPTIMA and ESPRIT studies:

All publications are to be approved by the TMG before submission for publication. The TMG will resolve problems of authorship and maintain the quality of publications.

Trial findings will be submitted to journal(s) that support open access publication within the time frame specified by the UCL policy. All publications will acknowledge funding sources.

For all publications, the TMG will either nominate a chairperson or approve an individual’s request to chair a manuscript writing committee. The chair will usually be the primary or senior author. The chairperson is responsible for identifying fellow authors and for determining with that group the order of authorship that will appear on the manuscript. The data derived from this study are considered the property of UCL.

The presentation or publication of any data collected by the participating investigators on patients entered into this study is under the direct control of the TMG. This is true whether the publication or presentation is concerned directly with the results of the study or is associated with the study in some other way. However, although individual participating investigators will not have any inherent right to perform analyses or interpretations or to make public presentations or seek publication of any of the data other than under the auspices of and with the approval of the TMG, they will be encouraged to propose analyses subject to the approval by the TMG.

**14.1 INTELLECTUAL PROPERTY**

All background intellectual property rights (including licences) and know-how used in connection with the study shall remain the property of the party introducing the same and the exercise of such rights for purposes of the study shall not infringe any third party’s rights.

All intellectual property rights and know-how in the protocol and in the results arising directly from the study, but excluding all improvements thereto or clinical procedures developed or used by each participating site, shall belong to UCLH. Each participating site agrees that by giving approval to conduct the study at its respective site, it is also agreeing to effectively assign all such intellectual property rights (“IPR”) to UCL and to disclose all such know-how to UCL.

Each participating site agrees to, at the request and expense of UCL execute all such documents and do all acts necessary to fully vest the IPR in UCL.

**15. REFERENCE LIST**

1. Cassetti I and Study 903E Team. The safety and efficacy of tenofovir DF in combination with lamivudine and efavirenz through 6 years in antiretroviral-naïve HIV-1-infected patients. HIV Clin Trials 2007; 8: 8164-8172.

2. Cook GJR, Lodge MA, Blake GM, et al. Differences in skeletal kinetics between vertebral and humeral bone measured by 18F-fluoride positron emission tomography in postmenopausal women. J Bone Miner Res 2000; 15: 763-769.

3. Frost ML, Cook GJ, Blake GM, et al. The relationship between regional bone turnover measured using 18F-fluoride Positron Emission Tomography and changes in BMD is equivalent to that seen for biochemical markers of bone turnover. J Clin Densitom 2007; 10: 46-54.

4. Frost ML, Cook GJR, Blake GM, Marsden PK, Fogelman I. The precision of 18F-fluoride positron emission tomography as used to measure regional bone turnover at the spine and hip. Osteoporos Int 2006; 17: 429-430.

5. Siddique M, Frost ML, Moore AEB, et al. Whole body (18) F-fluoride PET measurements of regional bone metabolism. Osteoporos Int 2012; 23: S577.

6. Messa C, Goodman WG, Hoh CK, Choi Y, Nissenson AR, Salusky IB, Phelps ME, Hawkins RA. Bone metabolic activity measured with positron emission tomography and 18F-fluoride ion in renal osteodystrophy: correlation with bone histomorphometry. J Clin Endo Metab 1993; 77: 949-955.

7. Piert M, Zittel TT, Becker GA, et al. Assessment of porcine bone metabolism by dynamic 18F-fluoride PET: correlation with bone histomorphometry. J Nucl Med 2001; 42: 1091-1100.

8. Cook GJR, Blake GM, Marsden PK, et al. Quantification of skeletal kinetic indices in Paget’s disease using dynamic 18F-fluoride positron emission tomography. J Bone Miner Res 2002; 17: 854-859.

9. Frost ML, Fogelman I, Blake GM, et al. Dissociation between global markers of bone formation and direct measurement of spinal bone formation in osteoporosis. J Bone Miner Res 2004; 19: 1797-1804.

10. Frost ML, Compston JE, Goldsmith D, et al. 18F-fluoride positron emission tomography measurements of regional bone formation in hemodialysis patients with suspected adynamic bone disease. Calcif Tissue Int 2013; 93: 436-447.

11. Frost ML, Cook GJ, Blake GM, et al. A prospective study of risedronate on regional bone metabolism and blood flow at the lumbar spine measured by 18F-fluoride positron emission tomography. J Bone Miner Res 2003; 18: 2215-2222.

12. Frost ML1, Siddique M, Blake GM, et al. Regional bone metabolism at the lumbar spine and hip following discontinuation of alendronate and risedronate treatment in postmenopausal women. Osteoporos Int 2012; 23:2107-2116.

13. Frost ML, Siddique M, Blake GM, et al. Differential effects of teriparatide on regional bone formation using 18F-fluoride positron emission tomography. J Bone Miner Res 2011; 26: 1002-1011.

14. Frost ML, Moore AE, Siddique M, et al. ¹⁸F-fluoride PET as a noninvasive imaging biomarker for determining treatment efficacy of bone active agents at the hip: a prospective, randomized, controlled clinical study. J Bone Miner Res 2013; 28:1337-1347.

15. Notes for guidance on Clinical Administration of Radiopharmaceuticals and Use of Sealed Radioactive Sources [online]. Available: <https://www.gov.uk/government/publications/arsac-notes-for-guidance> [Accessed 07 December 2016].

16. Pozniak A, Arribas JR, Gathe J, et al. Switching to tenofovir alafenamide, coformulated with elvitegravir, cobicistat, and emtricitabine, in HIV-infected patients with renal impairment: 48-week results from a single-arm, multicenter, open-label Phase 3 study. J Acquir Immune Defic Syndr 2016; 71:530-537.

17. Mills A, Crofoot G Jr, McDonald C, et al. Tenofovir Alafenamide Versus Tenofovir Disoproxil Fumarate in the First Protease Inhibitor-Based Single-Tablet Regimen for Initial HIV-1 Therapy: A Randomized Phase 2 Study. J Acquir Immune Defic Syndr 2015; 69:439-445.

18. Sax P, et al. Tenofovir alafenamide versus tenofovir disoproxil fumarate, coformulated with elvitegravir, cobicistat, and emtricitabine, for initial treatment of HIV-1 infection: two randomised, double-blind, phase 3, non-inferiority trials. Lancet 2015; 385: 2606-2615.

19. Orkin C, DeJesus E, Ramgopal M, et al. Switching from tenofovir disoproxil fumarate to tenofovir alafenamide coformulated with rilpivirine and emtricitabine in virally suppressed adults with HIV-1 infection: a randomised, double-blind, multicentre, phase 3b, non-inferiority study. Lancet HIV 2017; 4: 195-204.

20. Siddique M, Frost ML, Moore AE, Fogelman I, Blake GM. Correcting (18)F-fluoride PET static scan measurements of skeletal plasma clearance for tracer efflux from bone. Nucl Med Commun 2014; 35:303-310.

21. Rothman, KJ. No adjustments are needed for multiple comparisons. Epidemiology 1990; 1: 43-46.
